# Safety and efficacy of inhaled PEG-ADM in ARDS patients: a randomised controlled trial

**DOI:** 10.1186/s13054-025-05617-y

**Published:** 2025-10-23

**Authors:** Christian Karagiannidis, Danny F. McAuley, B. Taylor Thompson, Thomas Reimer, Kaweh Shakery, Sebastian Schmitz, Manuel Núñez Cortés, Roman Ullrich, Ferhat Meziani, Alain Mercat, Davide Chiumello, Frantisek Duska, Alain Combes

**Affiliations:** 1 ARDS and ECMO Centre Cologne-Merheim, University Witten/Herdecke, Ostmerheimer Strasse 200, 51109 Cologne, Germany; 2 Dentistry and Biomedical Science, Wellcome-Wolfson Institute for Experimental Medicine, School of Medicine, Queen’s University of Belfast, Belfast, UK; 3 Division of Pulmonary and Critical Care Medicine, Department of Medicine, Massachusetts General Hospital and Harvard Medical School, Boston, MA USA; 4 Pharmaceuticals Research & Development, Bayer AG, Berlin, Germany; 5 AUVA Trauma Centre, Vienna, Austria; 6 Service de Médecine Intensive-Réanimation, Nouvel Hôpital Civil, Hôpitaux Universitaires de Strasbourg, 1, Place de L’Hôpital, 67091 Strasbourg Cedex, France; 7 Département de Médecine Intensive Réanimation, CHU d’Angers, Université d’Angers, Angers, France; 8 Department of Health Sciences, University of Milan, Milan, Italy; 9 Department of Anesthesia and Intensive Care, ASST Santi Paolo E Carlo, San Paolo University Hospital Milan, Milan, Italy; 10 Coordinated Research Center On Respiratory Failure, University of Milan, Milan, Italy; 11 Dept. of Anaesthesia and Intensive Care Medicine, The Third Medical Faculty, Charles University, FNKV University Hospital, Prague, Czech Republic; 12 Institute of Cardiometabolism and Nutrition, Sorbonne Université, INSERM, UMRS_1166-ICANF-75013 Paris, France; 13 Service de Médecine Intensive-Réanimation, Institut de Cardiologie, APHP Sorbonne Université Hôpital Pitié-Salpêtrière, 75013 Paris, France

## Abstract

**Background:**

This study aimed to evaluate the safety and efficacy of inhaled pegylated adrenomedullin (PEG-ADM) for the management of acute respiratory distress syndrome in critically ill patients on mechanical ventilation.

**Methods:**

A Phase 2a/b randomised, double-blind, placebo-controlled multicentre trial was conducted. Patients with acute respiratory distress syndrome were assigned to receive PEG-ADM 960 μg or 1920 μg, or placebo. The primary endpoints were safety, efficacy, and ventilator-free survival at Day 28. Efficacy was assessed using ventilator-free survival and the clinical utility index, a composite endpoint that includes extravascular lung water index, oxygenation index, non-pulmonary Sequential Organ Failure Assessment score.

**Results:**

Ninety patients were randomised (PEG-ADM 960 μg: n = 29; PEG-ADM 1920 μg: n = 30; placebo: n = 31). Both dosages of PEG-ADM were well tolerated, with adverse event profiles similar to placebo. However, no significant efficacy was observed on the clinical utility index. Ventilator-free survival at Day 28 was lower in the PEG-ADM 960 μg group (52%) compared with the PEG-ADM 1920 μg (67%) and placebo (65%) groups. No significant differences were noted in overall mortality or the need for continued ventilation at Days 28 and 60.

**Conclusion:**

Inhaled PEG-ADM was well tolerated in patients with acute respiratory distress syndrome, but it did not improve clinical outcomes, which led to the early discontinuation after the first part of the trial for futility.

**Trial registration:**

ClinicalTrials.gov: NCT04417036 (date of registration: 4 June 2020).

**Supplementary Information:**

The online version contains supplementary material available at 10.1186/s13054-025-05617-y.

## Key message

**What is already known on this topic **- *summarise the state of scientific knowledge on this subject before you did your study and why this study needed to be done*

Acute respiratory distress syndrome (ARDS) is known to have a high 28-day mortality rate with no effective specific pharmacologic interventions currently available. PEGylated adrenomedullin (PEG-ADM) was hypothesised to reduce vascular permeability of lung endothelial cells and, therefore, improve pulmonary function and patient outcomes. An inhaled formulation was further developed to increase lung selectivity and reduce hypotensive systemic effects.

**What this study adds **-* summarise what we now know as a result of this study that we did not know before*

This Phase 2 randomised controlled trial investigated the safety and efficacy of inhaled PEG-ADM in ARDS patients on mechanical ventilation. Main efficacy outcomes included 28-day ventilator-free survival and the clinical utility index, which comprised the extravascular lung water index, change in oxygenation index, and change in non-pulmonary Sequential Organ Failure Assessment score. While PEG-ADM was well tolerated with a safety profile comparable to placebo, no meaningful clinical benefits were observed in both efficacy outcomes at either of the two investigated doses.

**How this study might affect research, practice or policy **-* summarise the implications of this study*

Further Phase IIB/IIA studies with inhaled ADM-PEG in ARDS are not warranted.

**Supplementary Information:**

The online version contains supplementary material available at 10.1186/s13054-025-05617-y.

## Introduction

While there has been significant progress in recent years, 28-day mortality in acute respiratory distress syndrome (ARDS) remains high (~ 30 to 45%) [1]. Treatment is largely supportive, with no effective specific pharmacological intervention currently available [2, 3]. Early resolution of lung oedema is associated with improved outcomes [4, 5], and reduction of fluid extravasation maybe a target to reduce extravasation of fluid and therefore progression of ARDS.

Adrenomedullin (ADM) is an endogenous peptide hormone associated with enhanced barrier function and reduced hyperpermeability of endothelial cells [6], with receptors highly expressed in lung endothelium [7, 8]. Wild-type ADM has a short half-life, so a PEGylated form (PEG-ADM; BAY 1097761) was developed in which ADM is bound to a 40 kDa polyethylene glycol polymer. Data from Phase 1 studies of PEG-ADM confirmed its prolonged half-life but revealed blood pressure-lowering effects after intravenous (IV) administration (Bayer AG, data on file). An inhaled formulation was, therefore, developed to increase lung selectivity and reduce the potential for hypotensive systemic effects. Here we presented data from a Phase 2 trial investigating the safety and efficacy of inhaled PEG-ADM in patients with ARDS.

## Methods

### Study design

This was a randomised, double-blind, placebo-controlled, multicentre Phase 2a/b clinical trial conducted in Austria, Czechia, France, Germany, Italy, Spain, and the United Kingdom (ClinicalTrials.gov: NCT04417036). Patients in Part A were randomised 1:1:1 to PEG-ADM 960 μg, PEG-ADM 1920 μg, or placebo (Supplementary Fig. 1). The study was initiated with the placebo and 960 μg arms, and safety was assessed by an unblinded independent data monitoring committee (DMC) after four patients were exposed to PEG-ADM 960 μg and again after 10 patients. The 1920 μg arm was opened when safety was confirmed by the DMC. The next 15 participants were recruited for the 1920 μg and placebo arms to assess the safety of the higher dose and, on confirmation of safety by the DMC, the 960 μg arm was reopened to allow parallel recruitment into all three study arms. Details of randomisation and blinding are presented in the Supplementary Methods. All site staff, patients, and the sponsor’s study team were blinded to treatment assignment.

Part B was a planned two-arm trial in which patients would be randomised, based on the results of Part A, to the PEG-ADM dosage with higher safety and efficacy or placebo (Supplementary Fig. 1). However, results from Part A led to termination of the study by the sponsor before Part B was initiated.

### Patients

Eligible patients were adults with ARDS (based on the Berlin definition [9]) requiring invasive mechanical ventilation. Patients were randomised within 48 h after meeting all diagnostic ARDS criteria for the first time. Before randomisation, patients were required to have hypoxaemia under ventilation, defined as minimum positive end-expiratory pressure [PEEP] ≥ 8 cm H_2_O, and partial pressure of oxygen in arterial blood/fraction of inspired oxygen [PaO_2_/FiO_2_] ≤ 300 mmHg continuously for ≥ 4 h, with values for ≥ 2 arterial blood gas (ABG) analyses measured during that time (last value obtained shortly before randomisation, ideally within ≤ 3 h). Key exclusion criteria included: any PaO_2_:FiO_2_ ratio > 300 mmHg within 4 h before randomisation; rescue therapy before baseline (e.g. inhalation of nitric oxide gas and/or inhalation of prostacyclin analogues, airway pressure release ventilation, or extracorporeal membrane oxygenation/extracorporeal carbon dioxide removal); expected survival < 24 h or expected duration of mechanical ventilation < 48 h; history of comorbidities requiring long-term oxygen use or other relevant lung conditions; and lung surgery, malignancy or diffuse alveolar haemorrhage from vasculitis. Based on a DMC recommendation, the protocol was amended after starting Part A enrolment to exclude patients diagnosed with coronavirus disease 2019 (COVID-19) pneumonia within 6 weeks before inclusion, as the heterogeneity introduced by these patients made it difficult to adequately evaluate drug effects. Patients with COVID-19 who had been enrolled before the amendment were included in the study database. Full inclusion and exclusion criteria are presented in Supplementary Table 1.

### Study procedures

PEG-ADM and placebo were administered three times daily using an Aerogen® Solo nebuliser device (Aerogen, Dangan, Ireland) via an endotracheal or tracheostomy tube. After randomisation, the first application of study treatment took place within 2 h. The recommended interval between inhalations was 8 h (permitted: 6–9 h), with the morning dose initiated between 5 and 8 am. Further details of study procedures are provided in the Supplementary Methods.

Treatment duration was up to 14 days or until extubation, after which it was assumed that treatment was no longer required. Total follow up was 90 days. All patient care was at the discretion of the treating physician, considering published recommendations on ventilator management, weaning criteria, and fluid management (see Supplementary Methods for details).

### Assessments and outcomes

At baseline, demographics and disease characteristics were recorded, along with ABG measurements, ventilator settings, extravascular lung water (EVLW), and non-pulmonary Sequential Organ Failure Assessment score (npSOFA) domains. Details of study assessments are presented in the Supplementary Methods including the detailed definition of AE´s and SAE´s.

ABGs, ventilator settings, and npSOFA domains were recorded daily from Day 2 to Day 7, as well as on Day 11 and the last day of treatment. ABGs and ventilator settings were also recorded 3 days after the last treatment if the patient was still intubated. Twelve-lead electrocardiogram was assessed on Days 2, 4, and 7, and on the last day of treatment. EVLW and fluid balance were assessed daily on Days 2–7. Vital status (including intubation/extubation status), hospitalisation status, vital signs, adverse events (AEs), and concomitant and rescue medication were monitored throughout the study.

Biomarkers related to the pathophysiology of ARDS were considered for phenotyping based on a latent-class-analysis as previously described by Calfee and colleagues (2018) [10]. These included inflammatory cytokines (interleukin-6, interleukin-8, soluble tumor necrosis factor receptor-1), angiopoetin-2 (a marker of vascular leakage/endothelial injury), soluble receptor for advanced glycation end products (a marker of epithelial injury), coagulation Protein C, and markers of inflammation and sepsis (bicarbonate, lactate, procalcitonin).

Treatment compliance during the study was defined as actual intake/protocol-planned intake, expressed as a percentage.

### Outcomes

For Part A, the primary outcome to assess efficacy was the clinical utility index (CUI), comprising three components: EVLW index (EVLWi), oxygenation index (OI), and npSOFA score. The overall primary endpoint of the study was ventilator-free survival (VFS) at Day 28 in Part B, and components of this endpoint (mortality and need for mechanical ventilation at Day 28) served as secondary endpoints and additional decision criteria in Part A. VFS, defined as participants alive and breathing without invasive mechanical ventilation support, was assessed at Days 28 and 60.

CUI and VFS were assessed in the overall population and in sub-populations based on previously described hyper-inflammatory and hypo-inflammatory sub-phenotypes [10]. Additional secondary endpoints included VFS at Day 60, overall mortality and need for ventilation at Days 28 and 60, and the components of the CUI. Safety and tolerability were assessed based on AEs and serious AEs (SAEs).

Due to the negative results in Part A, initially planned Part B was not und will not be conducted.

### Statistical analysis

The sample size calculation for Part A was based on two scenarios in which a slight improvement under placebo was assumed: the first represented a positive result for two of three endpoints (EVLWi and OI), while the second represented no mean differences between PEG-ADM and placebo. A sample size of 30 participants per arm would produce a sufficiently high probability (98%) of proceeding to Part B under the positive scenario and a very low probability (16%) of proceeding to Part B in the no effect scenario (Supplementary Table 2).

The safety analysis set was defined as all participants who received at least one inhalation of study medication. Efficacy was evaluated in the full analysis set, comprising all patients in the safety analysis set with baseline and post-randomisation data after first study drug administration available for at least one CUI component. The pharmacokinetic (PK) analysis set (PKS) was defined as all patients in the safety analysis set who provided at least one PK sample.

The three components of the CUI – EVLWi, OI and npSOFA – were incorporated into an analytical model to compare the effects of PEG-ADM versus placebo [11]. The CUI was calculated as the unweighted mean of EVLWi, OI, and npSOFA, each having a value of 0–1. For OI, the last measurement before first study drug administration was used as baseline. For EVLWi, the last measurement before or at the time of initiating first study drug administration was used as baseline. For npSOFA score, the first assessment on Day 1 was used as baseline. Values at baseline and on Days 2–7 were used for analysis. VFS was analysed based on a Bayesian analysis performed with a Beta (0.5,0.5) distribution as prior for each treatment arm. Details of the statistical methodology are presented in the Supplementary Methods.

## Results

### Patients and treatments

Of 98 patients screened, 90 were randomised to Part A between 8 July 2020 and 29 September 2022; they received study treatment and were included in the safety analysis set (PEG-ADM 960 μg, n = 29; 1920 μg, n = 30; placebo, n = 31) (Supplementary Fig. 3). All 90 patients were included in the full analysis set, and 51 patients (PEG-ADM 960 μg, n = 21; 1920 μg, n = 14; placebo, n = 16) were included in the PKS. Based on results from Part A, the study was terminated by the sponsor before Part B was initiated.

Baseline demographics and disease characteristics were similar across treatment arms, including age, body mass index, ARDS severity, vasopressor use, and ventilation parameters (Table 1). Consistent with the initial staggered approach to drug exposure and the aforementioned protocol amendment concerning COVID-19 patient exclusion, there were more patients with COVID-19 in the 960 μg arm (24%) than in the 1920 μg (3%) and placebo (13%) arms. This also contributed to an imbalance regarding the percentage of patients who recorded pneumonia as their ARDS trigger in the 1920 μg arm (30%) versus the 960 μg and placebo arms (66% and 71%, respectively). Requirement for renal replacement therapy was slightly more common in the 960 μg arm (28%) compared with the 1920 μg and placebo arms (20% and 19%, respectively).

**Table 1:** Baseline characteristics

	PEG-ADM 960 μg (n = 29)	PEG-ADM 1920 μg (n = 30)	Placebo (n = 31)
Sex, n (%)			
Male	21 (72)	23 (77)	20 (65)
Female	8 (28)	7 (23)	11 (35)
Age (years), mean (SD)	56.9 (16.8)	60.1 (17.8)	62.0 (11.3)
Body mass index (kg/m^2^), mean (SD)	29.3 (8.3)	27.7 (4.9)	28.4 (4.8)
Disease severity, n (%)			
Mild	3 (10)	5 (17)	6 (19)
Moderate	17 (59)	15 (50)	17 (55)
Severe	9 (31)	10 (33)	8 (26)
ARDS triggers, n (%)			
Aspiration of gastric acid	4 (14)	11 (37)	4 (13)
Pancreatitis	3 (10)	1 (3)	1 (3)
Pneumonia	19 (66)	9 (30)	22 (71)
Sepsis	3 (10)	9 (30)	4 (13)
COVID-19, n (%)	7 (24)	1 (3)	4 (13)
Vasopressor use, n (%)	27 (93)	28 (87)	26 (84)
Renal replacement therapy, n (%)	8 (28)	6 (20)	6 (19)
npSOFA score, mean (SD)	7.5 (2.8)	8.9 (3.5)	7.4 (3.4)
APACHE II score	19.9 (6.6)	23.5 (7.5)	20.5 (6.5)
Oxygenation index, mean (SD)	8.5 (6.0)	8.0 (3.5)^a^	6.6 (2.7)^b^
Ventilator parameters, mean (SD)			
Tidal volume (mL/kg)	6.7 (1.3)	6.8 (1.5)	6.8 (1.5)
PEEP (cm H_2_O)	11.6 (3.2)	10.7 (2.6)	10.7 (2.9)
Driving pressure	11.4 (3.2)^c^	12.2 (3.7)^d^	12.2 (4.0)^b^
Plateau pressure	23.1 (4.8)^c^	22.8 (4.5)^d^	23.1 (4.3)^b^

^a^n = 28; ^b^n = 29; ^c^n = 27; ^d^n = 23

APACHE II, Acute Physiologic Assessment and Chronic Health Evaluation II; ARDS, acute respiratory distress syndrome; COVID-19, coronavirus disease 2019; npSOFA, non-pulmonary Sequential Organ Failure Assessment; PEEP, positive end-expiratory pressure; PEG, polyethylene glycol; PEG-ADM, PEGylated adrenomedullin SD, standard deviation

In total, 81% of patients completed the treatment phase (Supplementary Fig. 3). Reasons for the end of the treatment were mainly death or in few patients adverse events or lost to follow up. All patients entered post-treatment follow-up, with 68% completing 90 days of total follow up. Mean (standard deviation [SD]) treatment duration was 10.1 (4.3) days with PEG-ADM 960 μg, 9.1 (4.0) days with PEG-ADM 1920 μg, and 10.5 (3.5) days with placebo. Mean treatment compliance was > 95% in all three treatment arms (PEG-ADM 960 μg, 96%; PEG-ADM 1920 μg, 96%; placebo, 98%).

### Safety

The overall incidence of AEs was similar across treatment arms, although pneumonia was more common with PEG-ADM 960 μg (10%) and 1920 μg (13%) versus placebo (3%) (Table 2). AEs considered to be drug-related by the investigator were reported in three patients receiving PEG-ADM 960 μg (eosinophilia; SAEs of decreased oxygen saturation and acute kidney injury), one patient receiving PEG-ADM 1920 μg (non-serious events of hyperlipasemia and hypotension). One patient receiving placebo showed also eosinophilia, deep vein thrombosis, and an SAE of pulmonary embolism. The number of patients experiencing AEs leading to treatment discontinuation was low and similar across study arms. The number of patients experiencing SAEs was also similar across treatment groups, although acute kidney injury was reported only in the PEG-ADM 960 μg arm (14%). Over the 90-day study period, 11 deaths were reported in the PEG-ADM 960 μg arm, compared with seven each in the placebo and PEG-ADM 1920 µg arms.

**Table 2:** Safety summary

Adverse events, n (%)	PEG-ADM 960 μg (n = 29)	PEG-ADM 1920 μg (n = 30)	Placebo (n = 31)
Any AE	24 (83)	27 (90)	27 (81)
Study drug-related AE	3 (10)	1 (3)	1 (3)
AE leading to discontinuation	2 (7)	3 (10)	2 (6)
Most frequent AEs^a^			
Acute kidney injury	6 (21)	5 (17)	0
Anaemia	1 (3)	3 (10)	5 (16)
Pneumonia	3 (10)	4 (13)	1 (3)
Hypertension	3 (10)	1 (3)	1 (3)
Septic shock	3 (10)	0	1 (3)
Hypotension	0	3 (10)	0
Any SAE	15 (52)	17 (57)	15 (48)
Study drug-related SAE	2 (7)	0	1 (3)
SAE leading to discontinuation	2 (7)	3 (10)	2 (6)
Most frequent SAEs^b^			
Acute kidney injury	4 (14)	0	0
Multiple organ dysfunction syndrome	1 (3)	1 (3)	2 (6)
Intestinal ischaemia	0	2 (7)	0
Pulmonary embolism	0	0	2 (6)
AE leading to death	11 (38)	7 (23)	7 (23)

AE, adverse event; PEG, polyethylene glycol; PEG-ADM, PEGylated adrenomedullin; SAE, serious adverse event

^a^AEs occurring in ≥ 10% of patients in any treatment arm

^b^SAEs occurring in ≥ 2 patients in any treatment arm

### Efficacy

**Fig. 1:**
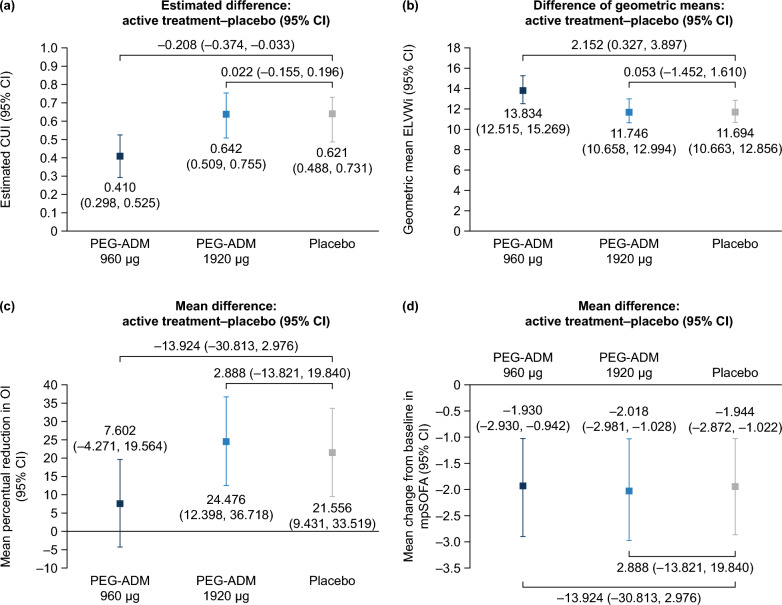
Clinical utility index (CUI) estimates for PEGylated adrenomedullin and placebo. (**a**) Overall CUI, (**b**) EVLWi mean treatment effect over Days 4–7, (**c**) mean percentual reduction in OI from baseline on Days 4–7, and (**d**) mean change in npSOFA from baseline at Day 7. CI, confidence interval; CUI, clinical utility index; EVLWi, extravascular lung water index; npSOFA, non-pulmonary Sequential Organ Failure Assessment score; OI, oxygenation index; PEG, polyethylene glycol; PEG-ADM, PEGylated adrenomedullin

CUI scores for the three treatment arms are shown in Fig. 1a. Estimated treatment–placebo differences (95% confidence interval [CI]) were low: –0.208 (–0.374, –0.033) for PEG-ADM 960 μg and 0.022 (–0.155, 0.196) for PEG-ADM 1920 μg. The posterior probability for a positive difference between active treatment and placebo was 1.2% and 60.3% for the 960 μg and 1920 μg doses, respectively.

The impact of treatment on the CUI components is shown in Fig. 1b–d. EVLWi remained high in all treatment arms, with no meaningful benefit from either PEG-ADM dose versus placebo. However, ELWI was in fact significantly higher in the low dose group compared to placebo, which may have affected the outcome of the study. Accordingly, CUI was significantly lower. No meaningful differences in OI and npSOFA improvement were observed between the treatment arms.

**Fig. 2:**
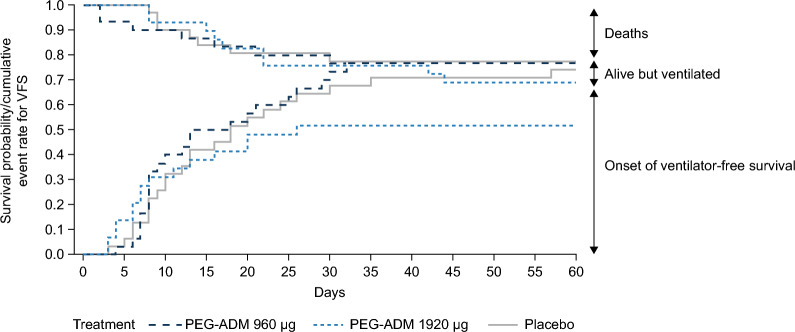
Combined Kaplan–Meyer curve for mortality and Aalen–Johansen curve for onset of ventilator-free survival at Day 60. PEG, polyethylene glycol; PEG-ADM, PEGylated adrenomedullin; VFS, ventilator-free survival

**Fig. 3:**
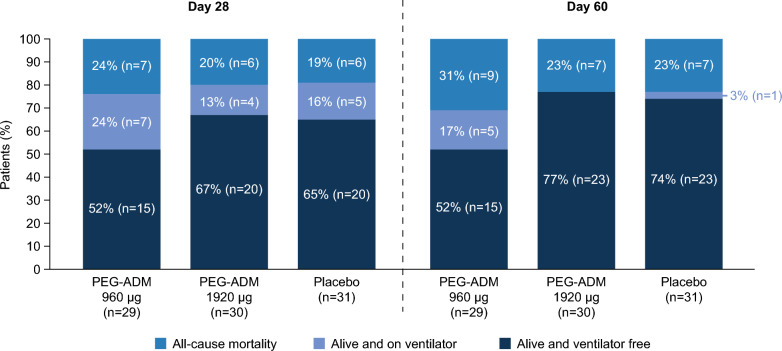
Mortality and ventilator status at Days 28 and 60. PEG, polyethylene glycol; PEG-ADM, PEGylated adrenomedullin

At Day 28, VFS was lower in the PEG-ADM 960 μg group (52%) compared with PEG-ADM 1920 μg and placebo (67% and 65%, respectively) (Fig. 2). This was primarily driven by the need for ventilation, as mortality was similar across the treatment arms. At Day 60, VFS had increased in the PEG-ADM 1920 μg and placebo groups and remained stable in the PEG-ADM 960 μg group (Figs. 2 and 3). At Day 90, all-cause mortality was 38% (n = 11) in the PEG-ADM 960 μg group, 23% (n = 7) in the PEG-ADM 1920 μg group, and 23% (n = 7) in the placebo group (Table 2). Up to Day 28, the mean numbers of ventilator-free days, overall hospital stay, and intensive care unit stay were similar across treatment arms (Supplementary Table 3).

### Analysis by inflammatory sub-phenotype

Categorisation by inflammatory sub-phenotype as described before [10], was possible for 26 patients in the PEG-ADM 960 μg arm (hyper-inflammatory, n = 9; hypo-inflammatory, n = 17), 28 patients in the PEG-ADM 1920 μg arm (n = 20 and n = 8, respectively), and 29 patients in the placebo arm (n = 8 and n = 21, respectively). CUI scores are shown in Supplementary Fig. 4. In patients with the hyper-inflammatory sub-phenotype, the estimated treatment–placebo differences (95% CI) were –0.186 (–0.537, –0.187) for PEG-ADM 960 μg and –0.081 (–0.384, 0.258) for PEG-ADM 1920 μg. The posterior probability for a positive difference between active treatment and placebo was 18.2% and 33.9% for the 960 μg and 1920 μg doses, respectively.

In patients with the hypo-inflammatory sub-phenotype, the estimated treatment–placebo differences (95% CI) were –0.164 (–0.399, 0.000) for PEG-ADM 960 μg and 0.158 (–0.114, 0.388) for PEG-ADM 1920 μg. The posterior probability for a positive difference between active treatment and placebo was 2.4% and 87.9% for the 960 μg and 1920 μg doses, respectively. VFS in both inflammatory sub-phenotypes was broadly similar to the overall population (see Supplementary Results for details). However, 24% of the patients had COVID-19 pneumonia, which was clearly not part of the hyper und hypo inflammatory stratification done before the pandemic.

## Discussion

This randomised, double-blind, placebo-controlled, Phase 2a/b study investigated the safety and efficacy of two dosages of inhaled PEG-ADM in patients with ARDS requiring invasive mechanical ventilation. Both PEG-ADM dosages were well tolerated, with an overall incidence of AEs similar to placebo and few serious and drug-related AEs. The study also supported the use of the CUI as a potential surrogate outcome parameter, providing a platform for research into targeted therapies for ARDS. However, no meaningful treatment effect of PEG-ADM was observed on CUI or its components. Although the higher dose showed a more favourable posterior probability of superiority, the observed effect sizes remained small. Given the lack of efficacy, the trial was terminated by the sponsor after Part A. Both 28-day and 60-day VFS were lower with the 960 μg dose than with the 1920 μg dose or placebo and, while VFS continued to improve after Day 28 in the latter two arms, it did not improve further with the lower dose. It is important to note, however, that the interpretation of VFS was limited by the small number of patients, particularly in the subgroups; only Part B would have had the statistical power to properly assess VFS. Furthermore, there was a slightly higher need for renal replacement therapy in the 960 μg group at baseline compared with the 1920 μg and placebo groups and, because of the protocol amendment excluding patients with COVID-19 during the study, more patients in the 960 μg dose group had COVID-19 infection at baseline.

This was the first RCT demonstrating the feasibility of using the CUI – including EVLW index (EVLWi), oxygenation index (OI), and npSOFA score – as the primary outcome in a Phase 2 trial, but it was not without limitations. As an efficacy measure, its sample size calculation assumed a marked change in EVLWi from baseline for PEG-ADM and a slight improvement over time for placebo, whereas little change from baseline was observed in our study for all treatment arms. Nonetheless, it was notable that the signal detected by the CUI concept developed for this study, assessed over the first 7 days of treatment, was consistent with VFS over 28 and 60 days (i.e. no apparent beneficial effect from the higher PEG-ADM dose over placebo and worse outcomes with the lower dose). There was, however, no correlation between selected CUI efficacy parameters and mortality, with the exception of npSOFA.

Similar to the overall effects, there was no favourable treatment effect on CUI for PEG-ADM in either inflammatory sub-phenotype. Also, subgroup analysis between mild vs moderate/severe ARDS or COVID vs non-COVID patients revealed no significant differences. In addition, there were only limited differences between PEG-ADM groups and placebo, given the insufficient power in Part A to detect VFS differences. Of note, the latent class analysis model used for classifying patients into sub-phenotypes has not been validated for patients with COVID-19. Most patients with COVID-19-related ARDS are classified as hypo-inflammatory, and previous studies have shown significantly higher mortality rates in those with hyper-inflammatory than hypo-inflammatory disease [12, 13]. However, it is possible that the choice of the model and imbalances caused by the small size of the patient sample may have contributed to these findings. Furthermore, the low dose group included more COVID-19 patients, which may have affected the outcome due to more severe lung failure.

PEG-ADM was hypothesised to reduce vascular permeability, potentially improving pulmonary function and oxygenation. However, no meaningful clinical effects of PEG-ADM were observed at either dosage, and there are several possible reasons for this. By the time study treatment was administered, the pathophysiological processes of ARDS may have been too advanced to be reversed by PEG-ADM. Furthermore, administration was closely monitored, but deposition for inhaled drugs cannot be routinely measured at the bedside, which would help to explain the results. It might be possible that inhaled administration did not lead to the predicted deposition and distribution in the lung. The heterogeneity of patients with ARDS, encompassing a variety of underlying aetiologies and severities of illness, can also make it challenging to detect a uniform treatment effect. Finally, the evaluation of sub-phenotypes might not have been adequately captured in the study population because of the limited sample size.

In conclusion, inhaled PEG-ADM was well tolerated in patients with ARDS receiving invasive mechanical ventilation. While the CUI was demonstrated to be a potentially useful surrogate outcome, there was no evidence of clinically meaningful efficacy for PEG-ADM at high dose over placebo and even a worse outcome with the lower dose, despite a higher rate of COVID-19 positive patients.

## Supplementary Information

Additional file1

## Data Availability

No datasets were generated or analysed during the current study.
