# Supplementary material for: Safety and efficacy of inhaled PEG-ADM in ARDS patients: a randomised controlled trial
Source: Crit Care. 2025 Oct 23;29:448. doi: 10.1186/s13054-025-05617-y (PMC12551264; doi:10.1186/s13054-025-05617-y)
Supplement: Supplementary file 1 — Additional file1 [file 13054_2025_5617_MOESM1_ESM.docx]

## Safety and efficacy of inhaled PEG-ADM in ARDS patients: a randomised controlled trial – online supplement

Christian Karagiannidis,^1^ Danny F McAuley,^2^ B. Taylor Thompson,^3^ Thomas Reimer,^4^ Kaweh Shakery,^4^ Sebastian Schmitz,^4^ Manuel Núñez Cortés,^4^ Roman Ullrich,^5^ Ferhat Meziani,^6^ Alain Mercat,^7^ Davide Chiumello,^8^ Frantisek Duska,^9^ Alain Combes^10^ for the SEAL Trial Investigators

### SEAL trial investigators

**Austria**

**Katharina Krenn, Roman Ullrich**: AUVA Trauma Centre, Vienna, Austria

**Philipp Eller**: Medizinische Universität Graz Klinische Abteilung für Innere Medizin, Graz

**Michael Joannidis**: Internistische Intensiv- und Notfallmedizin, Innsbruck

**Herbert Koinig**: Klinische Abteilung für Anästhesie und Intensivmedizin Mitterweg, Insbruck

**Czech Republic**

**Frantisek Duska**: Dept. of Anaesthesia and Intensive Care Medicine, Charles University, The Thirs Medical Faculty and FNKV University Hospital, Prague

**Roman Skulec, Vladimir Cerny**: Oddeleni intenzivni mediciny, Usti nad Labem

**Jan Berousek**: V Uvalu 84, Prague

**France**

**Alain Combes**: Hôpital de la Pitié-Salpétrière Service de Réanimation Médicale, Paris

**Frédéric Pene, Jean-Daneil Chiche**: Hôpital Cochin - APHP Service de Réanimation Médicale, Paris

**Alain Mercat**: CHU d'Angers Département de Médecine Intensive Réanimation, Angers

**Laurent Papazian, Sami Hraiech**: Hôpital Nord - APHM Service de Réanimation des Détresses Respiratoires et Infections Sévères, Marseille

**Nicolas Terzi**: Hopital Michallon - CHU Grenoble Alpes - Site Nord Service de Médecine Intensive-Réanimation, La Tronche

**Ferhat Meziani**: Hôpitaux Universitaires Nouvel Hôpital Civil, Strasbourg

**Germany**

**Christian Karagiannidis**: Lungenklinik - ARDS und ECMO-Zentrum Ostmerheimer**,** Cologne

**Ulf Günther**: Universitätsklinik für Anästhesiologie / Intensivmedizin / Notfallmedizin / Schmerztherapie**,** Oldenburg

**Tobias Welte**: Zentrum Innere Medizin Klinik für Pneumologie, Hannover

**Martin Witzenrath**: Charité - Campus Mitte Medizinische Klinik m. S. Infektiologie und Pneumologie, Berlin

**Norbert Weiler**: Univeristätsklinikum Schleswig-Holstein - Campus Kiel Klinik für Anästhesiologie und Operative Intensivmedizin, Kiel

**Marc Berger**: Klinik für Anästhesiologie und Intensivmedizin, Essen

**Italy**

**Giacomo Grasselli**: Anestesia e Terapia Intensiva Adulti, Milano

**Davide Chiumello**: Anestesia e Rianimazione, Milano

**Spain**

**Dr Antoni Betbese Roig**: Servicio de Medicina Intensiva, Barcelona

**Candelaria de Haro Lopez, Ana Ochagavia Calvo**: Àrea de Crítics Parc Taulí, Sabadell

**Ricardo Ferrer Roca**: Servicio de Medicina Intensiva Passeig de la Vall d'Hebron, Barcelona

**Rafael Mañez Mendiluce**: Servicio de Medicina Intensiva (Planta 1), L'Hospitalet de Llobregat, Barcelona

**UK**

**Jeremy Bewley**: Marlborough Street, Bristol

**Andrew Aswani**: Great Maze Pond, London

**Ingeborg Welters**: Prescot Street, Liverpool

**Matthew Wise**: Heath Park, Cardiff

**Robert Jackson**: Derriford Road, Crownhill, Plymouth

### Supplementary Methods

#### Randomisation and masking

Randomisation was executed via interactive web response system (IWRS) as stratified randomisation based on the defined respective participant characteristics. To accomplish random assignments, computer-generated randomisation lists were prepared by the sponsor or delegate and provided to the IWRS vendor. Before the study was initiated, the log-in information and directions for the IWRS were provided to each site. During the course of the study, the randomisation list was provided to the statistical analysis centre for the data monitoring committee (DMC) and its meetings. The pharmacokinetic evaluator (a separate function, with precautions taken to maintain blinding) was able to receive treatment information as far as necessary to perform the PK analysis.

After obtaining informed consent and fulfilling all prerequisites for randomisation, the participant was registered in the IWRS and assigned a unique nine-digit participant number. The first five digits identify the country and study site, and the last four digits were assigned to the participant of the specific site in increasing order. The sixth digit was used to distinguish between Part A and B.

Depending on dose, patients received one vial (960 μg or placebo) or two vials (1920 μg or placebo) per treatment, for inhalation, three times daily (TID). Vials contained lyophilizate for reconstitution, which could be active drug or placebo, and were indistinguishable.

#### Study procedures

The medical devices used in this study were the Aerogen^®^ Solo nebuliser, Aerogen^®^ T-Piece, and Aerogen^®^ USB Controller (Aerogen, Dangan, Ireland). The nebuliser device was located in the ventilation circuit, in the inspiratory limb of the ventilation circuit before the Y-piece. It was recommended to use an extension tube between the Y-piece and endotracheal tube to keep the distance (and dead space) as small as possible. Extensions were to be straight and not exceed 15 cm in length wherever possible. When a coaxial ventilation tubing (or other ventilation circuit tubing that would not allow nebuliser placement in the inspiratory limb before the Y-piece) was used, the device was placed between the used ventilatory circuit tubing and the endotracheal tube of the participant. Use of an active humidifier was encouraged, but heat and moisture exchangers and filters could not be placed between the nebuliser device and the participant during inhalations.

The number of doses on Day 1 was dependent on the time of the first dose administration (i.e. it could be <3 doses on Day 1 if the first dose was given late in the day). If the first dose on Day 1 was outside the specified time window for a morning dose (5–8 am), all study activities and assessments otherwise triggered by the morning dose on Day 1 were performed accordingly after this first dose. The first dose in the morning of Day 2 was then initiated between 5 and 8 am, even if this meant that the interval to the last dose on Day 1 was >9 hours. However, a minimum interval of 6 hours was always observed.

On days when weaning procedures and/or spontaneous breathing trials were planned, study administration took place during times of controlled ventilation wherever possible, even if this violated the planned time window (the minimum interval of 6 hours between two study intervention applications was always observed). During spontaneous breathing attempts, study intervention was avoided, and no study intervention was administered after a participant was extubated. If subsequent reintubation occurred on or before Day 14, study intervention was restarted, but no study interventions were administered after Day 14 (end of treatment period), regardless of the number of prior extubations/reintubations or temporary discontinuations.

#### Recommendations on ventilator management

A modified, simplified version of the acute respiratory distress syndrome (ARDS) Network lung protective lower tidal volume strategy was recommended in this trial [1]. For participants who remained hospitalised and on mechanical ventilation, ventilator management and weaning recommendations were implemented up to Day 60 of hospitalisation.

1. Any mode of ventilation capable of delivering the prescribed tidal volume (6 mL/kg predicted body weight [PBW], ±2 mL/kg) could be used, provided the tidal volume target was monitored and adjusted appropriately.
2. Tidal Volume (Vt) Goal: 6 mL/kg PBW. PBW was calculated from age, sex, and height (heel to crown) according to the following equations used previously in ARDS studies [2,3]:

$$Males:PBW \left( kg \right)=50+2.3 \times\left[ height \left( inches \right)-60 \right]$$

$$or PBW \left( kg \right)=50+0.91 \times\left[ height \left( cm \right)-152.4 \right]$$

$$Females:PBW \left( kg \right)=45.5+2.3 \times\left[ height \left( inches \right)-60 \right]$$

$$or PBW \left( kg \right)=45.5+0.91\times[height \left( cm \right)-152.4]$$

3. Inspiratory plateau pressure (Pplat) was measured according to the intensive care unit (ICU) routine (recommended: at least every 4 hours and after changes in Vt and positive end expiratory pressure [PEEP]).
4. If Pplat was >30 cm H_2_O, Vt was reduced to 5 mL/kg and then to 4 mL/kg PBW if necessary to decrease Pplat to ≤30 cm H_2_O.
5. If Vt was <6 mL/kg PBW and Pplat <25 cm H_2_O, Vt could be raised by 1 mL/kg PBW to a maximum of 6 mL/kg (e.g., if the investigator considered the pH too acidotic).
6. In the event of severe dyspnoea (defined as >3 double breaths per minute or airway pressure at or below PEEP level during inspiration), Vt was raised to 7 or 8 mL/kg PBW if Pplat remained <30 cm H_2_O. If Pplat exceeded 30 cm H_2_O with Vt of 7 or 8 mL/kg PBW, then Vt was reverted to a lower level and additional sedation was considered.
7. If pH was <7.15, Vt could be raised and the Pplat limit suspended (not required).
8. The oxygenation target was 55 mmHg < partial pressure of oxygen in arterial blood (PaO_2_) < 80 mm Hg or 88% < oxygen saturation (SpO_2_) < 95%. When both PaO_2_ and SpO_2_ were available simultaneously, the PaO_2_ criterion took precedence.
9. Minimum PEEP per Berlin criteria was 5 cm H_2_O at time of diagnosis.
10. The minimum required PEEP (as set on the ventilator) during the screening period was 8 cm H_2_O to confirm the diagnosis before randomisation. Early in the disease course, ventilator PEEP values of 8–12 cm H_2_O for mild ARDS (PaO_2_/fraction of inspired oxygen [FiO_2_] ratio 201–300 mmHg) and PEEP values of 10–15 cm H_2_O for moderate or severe ARDS (PaO_2_/FiO_2_ ratio ≤200 mmHg) were recommended.
11. Deviation from the above-described PEEP strategy could be considered, especially in the following situations:
    - If there was clinical concern that the use of high PEEP may be worsening oxygenation (e.g. oxygenation worsened with PEEP increases) at a FiO_2_ ≥0.5 for >2 hours.
      - If oxygenation worsened or was unchanged at the lower level of PEEP, PEEP was raised back to the previous level.
      - If oxygenation improved, the treating clinicians could choose to leave the PEEP at the lower level.
    - If there was clinical concern that the use of high PEEP may be worsening haemodynamics or hypercarbia/acidaemia.
      - If hypotension, high plateau pressure (>30 cm H_2_O), falling static total respiratory system compliance (or increasing driving pressure on constant Vt), and/or severe acidosis (pH <7.15) were present despite low tidal volume, adequate fluid management, and/or respiratory rate adaption, lower PEEP could be used. If the physiological parameters of concern improved, the treating physician could consider returning to a higher PEEP, or may choose to leave PEEP at the lower level, depending on the clinical situation.
    - Lower PEEP may also be used if a study participant develops a pneumothorax, or is deemed at high risk for barotrauma (e.g. known multiple pulmonary cysts or bullae).
12. There were no specific rules for respiratory rate, but the rate could be increased incrementally at the investigator’s discretion to a maximum set rate of 35 if pH was <7.20–7.30.
13. There were no specific rules about inspiration/expiration ratio, but it was recommended that the duration of inspiration be the same of less than the duration of expiration.
14. Bicarbonate was optional if pH was <7.30. The investigator could also use the HCO_3_ value in decision making (e.g. if below 20 mmol/L).
15. Changes in more than one ventilator setting driven by measurements of PaO_2_, pH, and Pplat could be performed simultaneously if necessary.

Participants could be removed from the 6 mL/kg tidal volume ventilation requirement at the discretion of the clinical team caring for the participant if they developed neurologic conditions in which hypercapnia would be contraindicated (e.g. intracranial bleeding, cerebral oedema, mass effect [midline shift on computed tomography scan], papilloedema, intracranial pressure monitoring, or fixed pupils).

To help ventilation and administration of study intervention, in the event of evidence or suspicion of a relevant airway obstruction (e.g. mucoid impaction/mucus

plugging, extensive airway secretion), clearing of the airways (e.g. by suctioning or bronchoscopy) was considered according to the standard practices of the ICU.

#### Recommendations on weaning from ventilator

All patient care was per the treating physicians, considering the aspects outlined below. The following recommendations for standardised weaning are based on precedence set in the ROSE trial [1].

- Participants could be assessed for weaning readiness criteria twice a day:
  - FiO_2_ ≤0.50 and PEEP ≤8 cm
  - Values of both PEEP and FiO_2_ lower than values from previous day
  - Systolic arterial pressure ≥90 mm Hg without vasopressor support (≤5 μg/kg/min dopamine was not considered a vasopressor for this assessment).
- If all three weaning criteria were met, all neuromuscular blocking agents were discontinued. After the neuromuscular blocking agent had worn off and the participant was having spontaneous respirations, a trial of up to 120 minutes of spontaneous breathing with FiO_2_ <0.5 was initiated using any of the following approaches:
  - Pressure support <5 cm H_2_O, PEEP <5 cm H_2_O
  - CPAP <5 cm H_2_O
  - T-piece
  - Tracheostomy mask
- Tolerance was monitored using the following criteria:
  - SpO_2_ ≥90% and/or PaO_2_ ≥60 mmHg
  - Mean spontaneous tidal volume ≥4 mL/kg PBW (if measured)
  - Respiratory rate ≤35/min
  - pH ≥7.30 (if measured)
  - No respiratory distress (defined as two or more of the following):
    - Heart rate ≥120% of the rate at 6 am (≤5 min at >120% may be tolerated)
    - Marked use of accessory muscles
    - Abdominal paradox
    - Diaphoresis
    - Marked subjective dyspnoea.

If any of the tolerance goals were not met, the ventilator was reverted to the previous ventilator settings or to pressure support (PS) + 10 cm H_2_O with PEEP and FiO_2_ as the previous setting, and the patient was reassessed for weaning the next morning.

The clinical team caring for the participant were permitted to change the mode of support during spontaneous breathing (PS = 5, CPAP, tracheostomy mask, or T‑piece) at any time.

If the five tolerance criteria listed above for spontaneous breathing were met for ≥30 minutes, the clinical team could decide to extubate. The spontaneous breathing trial could, however, continue for up to 120 minutes if tolerance remained in question. If any of the tolerance criteria were not met during unassisted breathing (or when 120 minutes had passed without clear tolerance), then the ventilator settings that were in use before the attempt to wean were restored, and the participant was reassessed for weaning on the next day.

The definition of unassisted breathing (i.e. not on ‘invasive mechanical ventilation’) was:

- Extubated with face mask, nasal prong oxygen, or room air, OR
- Tracheostomy mask breathing, OR
- Extubated, with continuous pulmonary airway pressure (CPAP) ≤5 without pressure support/invasive mechanical ventilation assistance
- Extubated with CPAP or tracheostomy mask breathing solely for sleep apnoea management
- Extubated with use of any high-flow oxygen system

Participants were considered to have completed the study ventilator procedures in the vent of death, ICU discharge, and/or hospital discharge. If a participant required positive pressure ventilation after a period of unassisted breathing, the study ventilator procedures resumed unless the participant was discharged from the ICU or hospital. The endpoint “Ventilator free days” was assessed only for days off invasive mechanical ventilation after the last extubation in the defined timeframe. Days off ventilation between periods of invasive mechanical ventilation were not considered. For the assessment of whether a participant was free from ventilation at Day 28, follow-up was continued until Day 35 and only those participants who did not require reinitiation of invasive mechanical ventilation during this timeframe were regarded as successfully being off ventilation at Day 28.

#### Recommendations on fluid management

All patient care was per the treating physicians, considering the aspects outlined

below.

Fluid management during shock was unrestricted. For participants not in shock, however, a conservative fluid approach was recommended for all participants. This conservative fluid management approach represented a simplification of the algorithm used in the randomised, controlled FACTT study in patients with ARDS [4]. The recommendation was based on the findings from the FACTT study that conservative fluid management increased ventilator free days and, therefore, had a beneficial effect on the clinical outcomes of ARDS patients. This simplified modification of the conservative fluid management protocol from the FACTT study has also been used in the ROSE study [1]. It was recommended for all participants until the end of invasive mechanical ventilation or Day 7, whichever occurred first.

1. Discontinue maintenance fluids
2. Continue medications and nutrition
3. Manage electrolytes and blood products per usual practice
4. For shock, any combination of fluid boluses (recommended fluid bolus: 15 mL/kg crystalloid, rounded to nearest 250 mL, or 1 unit packed red cells or 25 grams albumin) and vasopressor(s) could be used to achieve mean artery pressure ≥60 mmHg as quickly as possible. Vasopressors were weaned as quickly as tolerated beginning 4 hours after blood pressure had stabilised
5. Diuretic therapy was withheld during renal failure (defined as dialysis dependence, oliguria with serum creatinine >3 mg/dL, or oliguria with serum creatinine 0–3 with urinary indices indicative of acute renal failure) and until 12 hours after the last fluid bolus or vasopressor was given

For participants without a central venous catheter, no fluid gain over the first 7 days was recommended after participants’ blood pressure had stabilised. Stable blood pressure was defined as no requirement for either vasopressors or a fluid bolus to support blood pressure for ≥12 hours. Fluid management is summarised in **Supplementary Table 4**.

#### Assessments and outcomes

Arterial blood gas measurements recorded during the study were partial pressure of oxygen, partial pressure of carbon dioxide, pH, bicarbonate, base excess, and lactate. Recorded ventilator settings were ventilator mode, fraction of inspired oxygen, tidal volume, actual respiratory rate, positive end expiratory pressure, mean airway pressure, peak airway pressure, and plateau pressure. Extravascular lung water (EVLW) was measured via PiCCO system (Pulsion Medical Systems, Getinge AB, Göteborg, Sweden) in accordance with the manufacturer’s instructions, with values recorded directly after daily device calibration had taken place. EVLW was indexed to individual patients’ predicted body weight to obtain the EVLWi. Baseline disease characteristics included Acute Physiologic Assessment and Chronic Health Evaluation (APACHE) II score, 12-lead electrocardiogram, fluid balance, vasopressor use, local and central laboratory assessments, intubation and ventilation status, vital signs, concomitant and rescue medications, and proning procedure.

Definition of AE and SAE


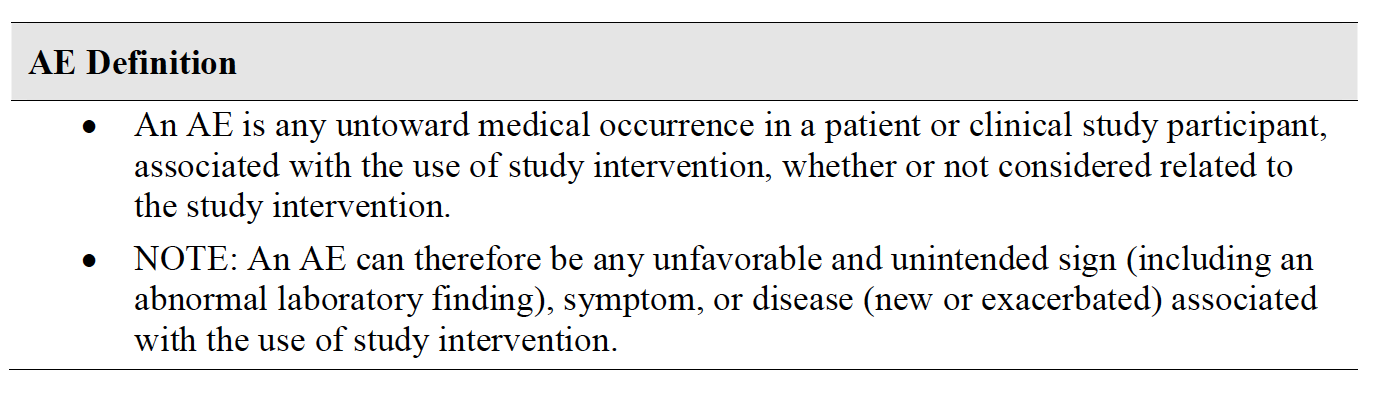


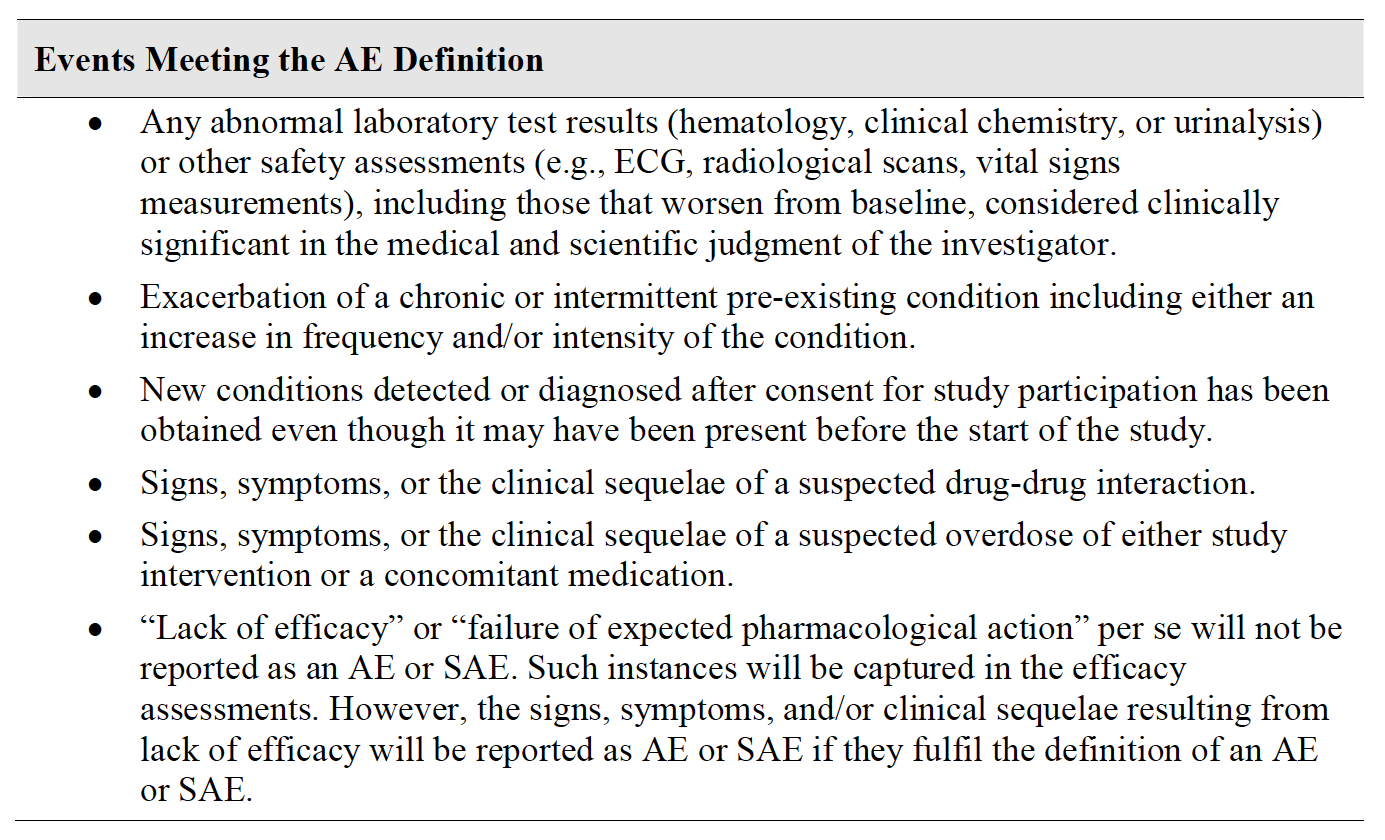


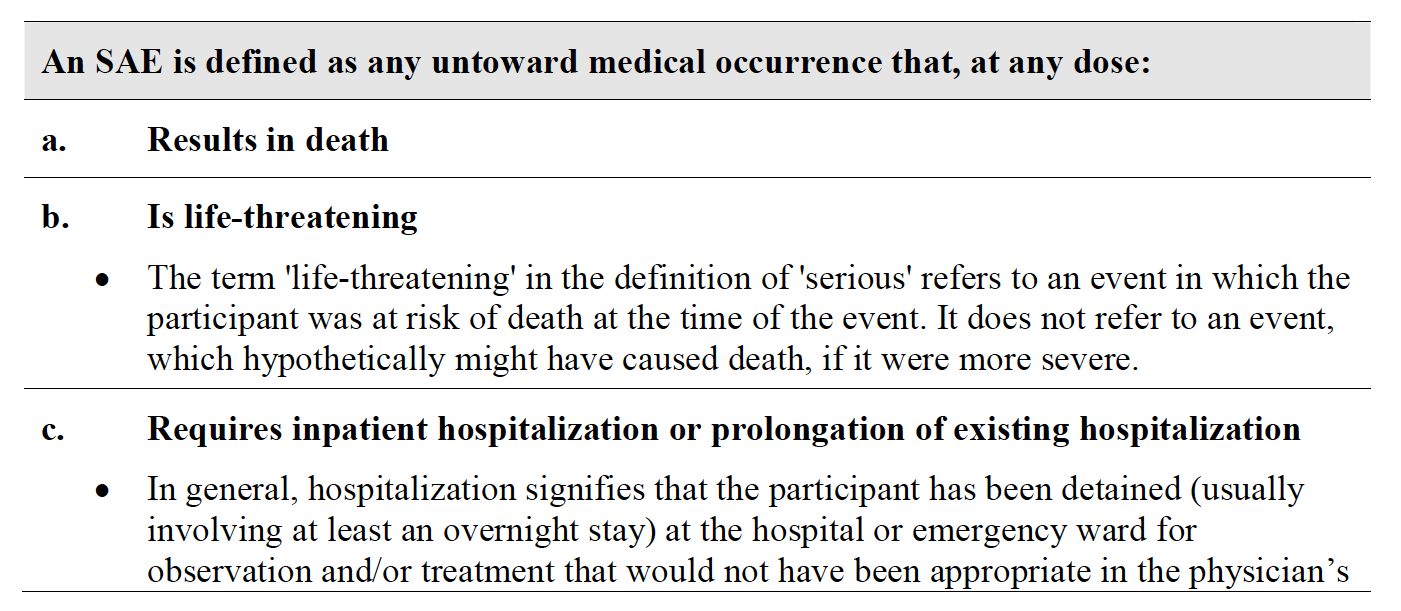


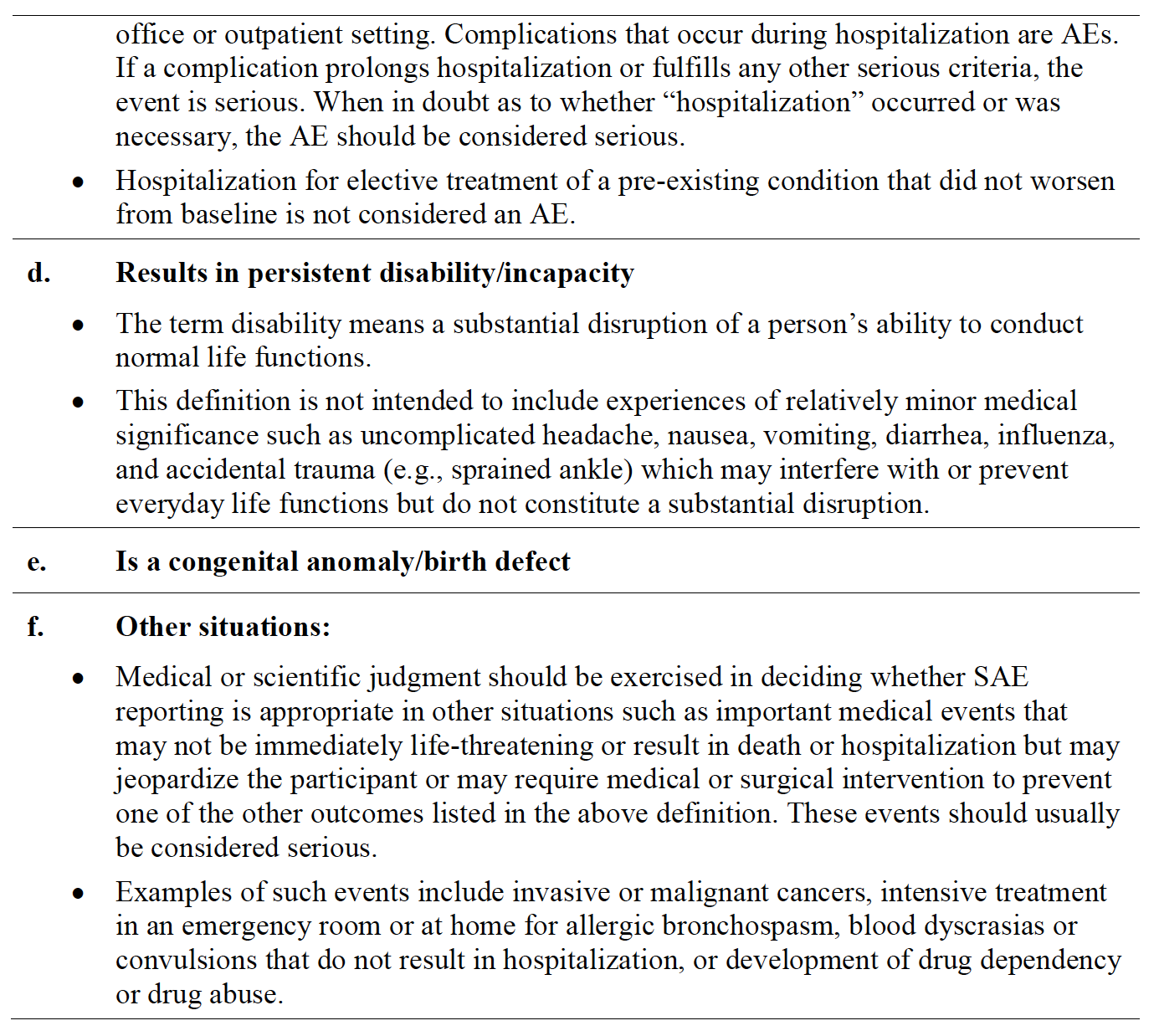


#### Statistical analysis

For the sample size calculation in Part A, the positive scenario involved a geometric mean change in EVLWi after treatment of 7 mL/kg for PEGylated adrenomedullin (PEG-ADM) and 12 ml/kg for placebo, and a mean relative change in OI of 40% and 14%, respectively.

For the main analysis of VFS, a Bayesian analysis was performed with a Beta (0.5,0.5) distribution as prior for each treatment arm. The resulting posterior distribution for each treatment arm was then a Beta (0.5+k,n-k+0.5) distribution. Point estimates and 95% credible intervals for the difference in proportions between each of the active treatments and placebo were determined based 10000 drawn posterior samples. Furthermore, the corresponding posterior probability was provided for superiority of each of the active treatment arms vs placebo.

The CUI was calculated as unweighted mean of three utilities:

$$CUI=\frac{(U_{EVLWi}+U_{OI}+U_{npSOFA})}{3}$$

The utilities are defined as follows:

$$U_{EVLWi}= \left\{ \begin{matrix} 0 & \leftrightarrow& \mu_{EVLW}>14 \\ \frac{14-\mu_{EVLW}}{7} & \leftrightarrow& 7\leq\mu_{EVLW}\leq14 \\ 1 & \leftrightarrow& \mu_{EVLW}<7 \end{matrix} \right.$$

$$U_{OI}=\left\{ \begin{matrix} 0 & \leftrightarrow& \delta_{OI}<0\% \\ \frac{\delta_{OI}}{40\%} & \leftrightarrow& 0\%\leq\delta_{OI}\leq40\% \\ 1 & \leftrightarrow& \delta_{OI}>40\% \end{matrix} \right.$$

$$U_{npSOFA}\left\{ \begin{matrix} 0 & \leftrightarrow& \delta_{npSOFA}>1 or mean npSOFA>11 at Study Day 7 \\ 0.1 & \leftrightarrow& -1\leq\delta_{npSOFA}\leq1 and mean npSOFA\leq11 at Study Day 7 \\ 1 & \leftrightarrow& \delta_{npSOFA}<-1 and mean npSOFA\leq11 at Study Day 7 \end{matrix} \right.$$

Where μ_EVLW_ is the mean EVLWi treatment effect over Study Days 4 to 7 of EVLWi, δ_OI_ is the mean percentual reduction in OI from baseline over Study Days 4 to 7, and μ_npSOFA_ is the mean absolute change in npSOFA from baseline at Study Day 7.

To compute μ_EVLW_, δOI and μ_npSOFA_, a Bayesian approach with non-informative priors was applied using Markov chain Monte Carlo (MCMC) simulation where log(EVLWi), OI percentual reduction from baseline, and npSOFA absolute change from baseline were assumed to be normally distributed response variables. It was assumed that correlation within a single patient was described fully by the random patient effect, which was equivalent to assuming a common correlation between all measurements of a single patient.

The following ANCOVA-type repeated measurements model was used, with treatment (${trt}_{k}$ in the formula for the k^th^ treatment), time point (${time}_{t}$ in the formula for the t^th^ time point), and treatment-by-time interaction as fixed effects, as well as participant ($P_{i}$ in the formula for the i^th^ participant) as a random effect. Baseline mean ($Y_{i}^{component}$ in the formula, used as normalised value with mean zero) was used as a covariate, ${CUI}_{ikt}^{component}$ denotes the CUI components log(EVLWi), OI percentual reduction from baseline, and npSOFA change from baseline, resulting in the following model equation:

$${CUI}_{ikt}^{component}=\mu+\beta\cdot Y_{i}^{component}+{trt}_{k}+{time}_{t}+\left( trt*time \right)_{kt}+P_{i}+\varepsilon_{ikt}$$

$P_{i}$ and $\varepsilon_{ikt}$were assumed as normally distributed random variables with mean 0 and standard deviation τ and σ, respectively. β describes the baseline covariate effect.

Under missing-at-random assumption, data of CUI components collected at baseline and at Day 2 up to Day 7 were analysed by the repeated measurements model mentioned above. Imputation of missing data was conducted using a fully conditional specification method with regression, with five imputation data sets created and used for the following analysis. Imputation values of npSOFA were rounded to the nearest integer value. Normalising of baseline CUI values was done after multiple imputation and rounding of npSOFA values. All kept posterior samples from all imputation data sets were used to compute point estimates and confidence intervals for the CUI analysis.

To calculate $U_{npSOFA}$, the npSOFA score on Day 7 was modelled assuming normal distribution and applying an ANOVA and model equation with parameters defined as above:

$${np}_{ik}^{D7}=\mu+{trt}_{k}+\varepsilon_{ik}$$

The Bayesian approach analogue to the npSOFA absolute change to baseline was applied, adapting the MCMC analysis and programming statements accordingly. The multiple imputation data set for npSOFA absolute change from baseline was used after rounding to obtain individual npSOFA values on Day 7.

With the MCMC posterior samples of the previously described four different MCMC simulations, the mapping to utilities and the determination of CUI and its posterior distribution was performed for $U_{EVLWi}$, $U_{OI}$, and $U_{npSOFA}$ using the following formulae:

$$\mu_{{ELVW}_{kt}}=exp(\mu+{trt}_{k}+{time}_{t}+\left( trt*time \right)_{kt})$$

$$\delta_{{OI}_{kt}}=(\mu+{trt}_{k}+{time}_{t}+\left( trt*time \right)_{kt})$$

$$\delta_{npSOFA}=(\mu+{trt}_{k}+{time}_{D7}+\left( trt*time \right)_{kD7})$$

The probability distribution of the CUI $U_{EVLWi}$, $U_{OI}$, and $U_{npSOFA}$ were then obtained. Assuming that a study arm was considered safe by safety assessments, a decision to continue the study arm was given if the CUI for the respective arm was larger than the CUI for placebo with ≥80% posterior probability and further efficacy evaluations outlined in subsequent sections showed favourable treatment effect compared with placebo.

Based on the hypothesis that latent hypo-/hyperinflammatory phenotypes exist, potentially having different underlying pathophysiologic mechanisms and responding differently to assigned study intervention, a latent class analysis (LCA) was applied [5].

Baseline clinical and plasma biomarker data, without consideration of intervention or outcomes, were used to identify phenotypes using LCA. Baseline demographics, available baseline clinical data, and baseline biomarkers were used as class-defining variables. This model was derived from variables previously used in an LCA of data from the FACTT study, and variables were prioritised according to observed-group differences in FACTT [5].

Models ranging from one to four classes were estimated to identify the optimal number of classes. From these four models, the best fit was evaluated using Bayesian Information Criteria (BIC). To check the robustness of the model decision, the analysis was performed 100 times using different seeds. The best-fitted model (based on BIC) was also tested against the other class-models using log-likelihood tests and adjusted p-values (Bonferroni-Holm). Before modelling, variables were examined for their distribution, and continuous variables with skewed distributions were log-transformed. To estimate model parameters, all continuous variables were scaled to a mean of 0 and standard deviation of 1.

**Supplementary Results**

#### Analysis by inflammatory sub-phenotype

Results for VFS in the inflammatory sub-phenotypes were broadly similar to those in the overall population, with VFS increasing from Day 28 to Day 60 in the PEG-ADM 1920 μg and placebo arms and remaining stable in the PEG-ADM 960 μg arm (**Supplementary Figure 5**). VFS was higher in hypo-inflammatory patients (Day 28: 53–76%; Day 60: 53–88%) than in hyper-inflammatory patients (25–44% and 44–70%, respectively).

### Supplementary figures and tables

**Supplementary Figure 1**. Planned study design for 182 patients until the first Interim analysis with a 1:1 randomization.

**
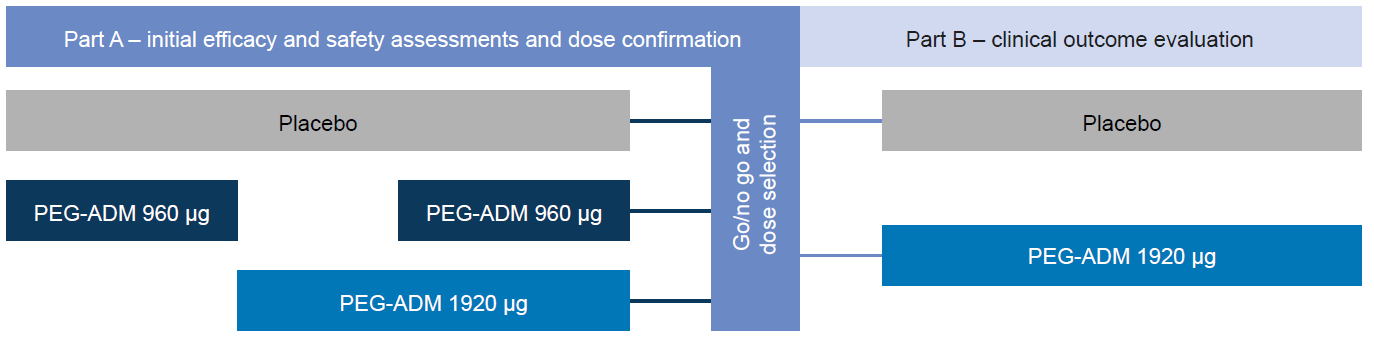
**

PEG, polyethylene glycol; PEG-ADM, PEGylated adrenomedullin.

**Supplementary Figure 2**. Decision tree for progression to Part B


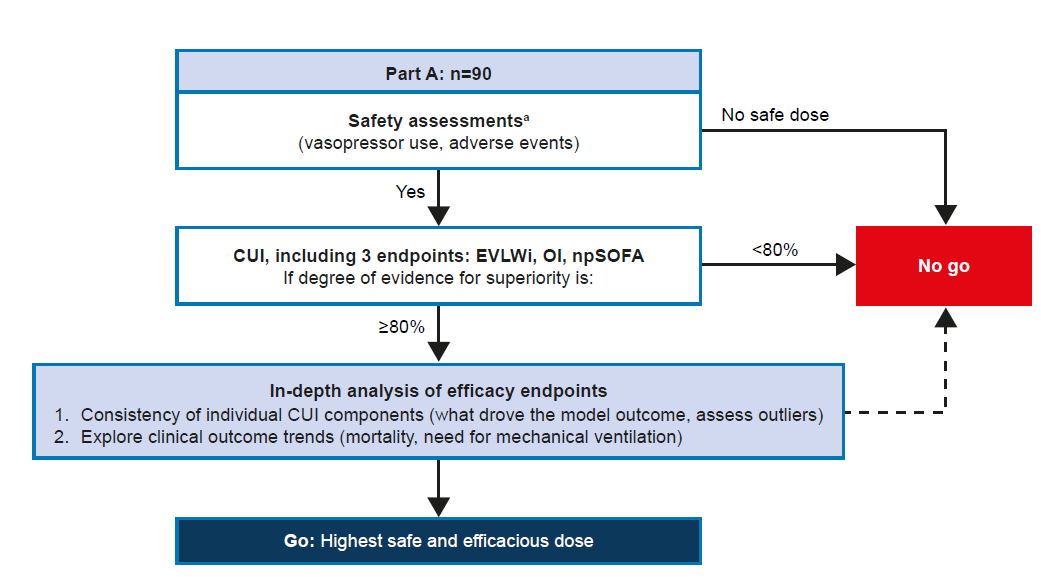


^a^Individual stop criteria in place; Data Monitoring Committee assessment at pre-defined time points during Part A and at end of Part A.

^b^Alive and not ventilated at Study Day 28.

CUI, Clinical Utility Index; EVLWi, extravascular lung water index, npSOFA, non-pulmonary Sequential Organ Failure Assessment; OI, oxygenation index.

**Supplementary Figure 3**. Participant flow chart


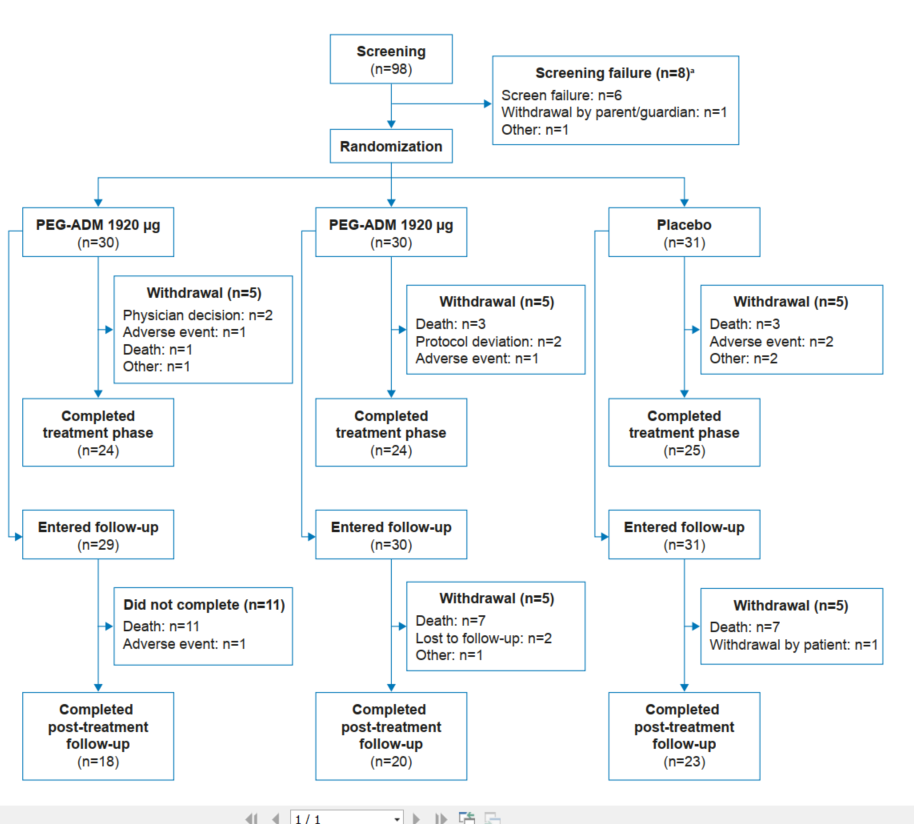


**PEG-ADM** 960 µg
(N=30)

^a^ Screening failures were patients for whom informed consent was obtained from the patient or legal representative, but who subsequently did not meet the study eligibility criteria.

PEG, polyethylene glycol; PEG-ADM, PEGylated adrenomedullin.

**Supplementary Figure 4**. CUI estimates for PEG-ADM and placebo in patients categorised as (a) hyper-inflammatory and (b) hypo-inflammatory


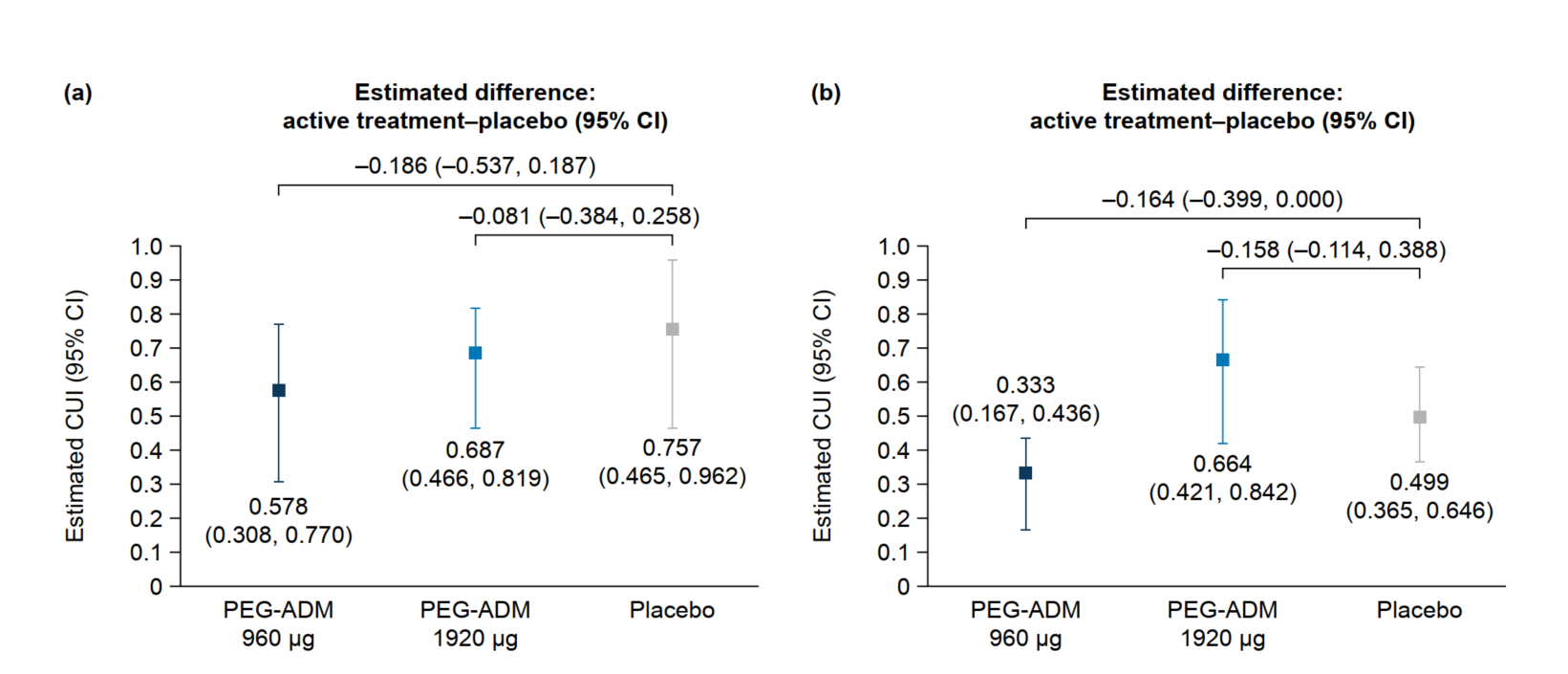


CI, confidence interval; CUI, clinical utility index; PEG, polyethylene glycol; PEG‑ADM, PEGylated adrenomedullin.

**Supplementary Figure 5**. Mortality and ventilator status at Days 28 and 60 in patients categorised as (a) hyper-inflammatory and (b) hypo-inflammatory


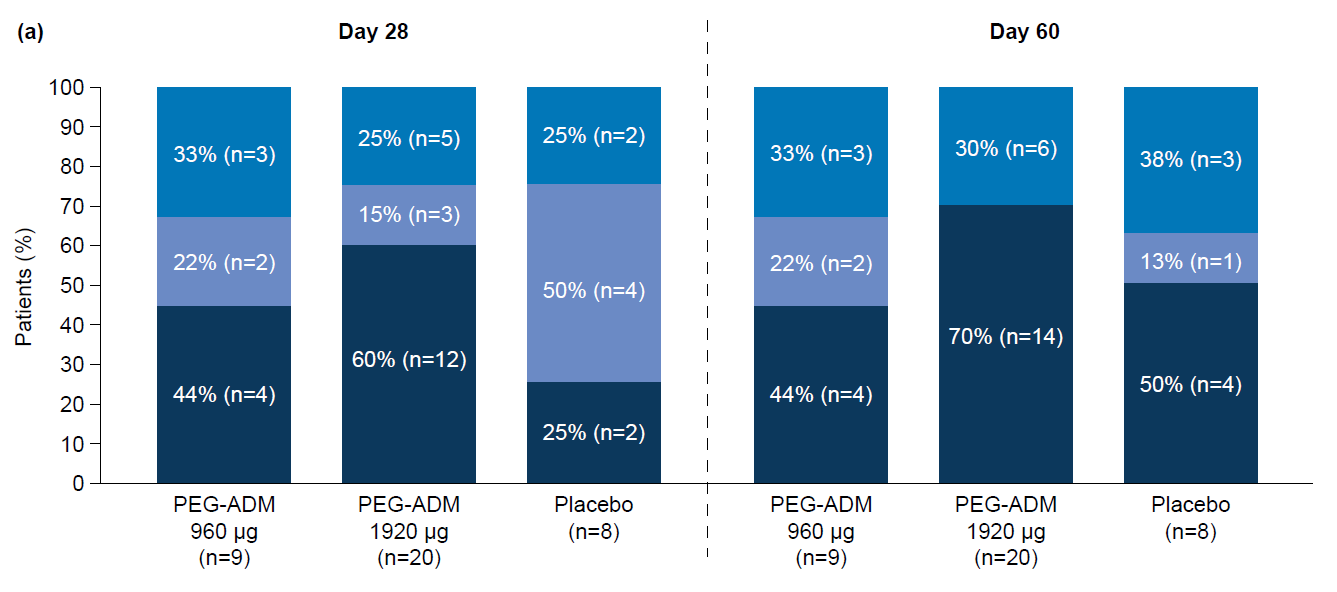


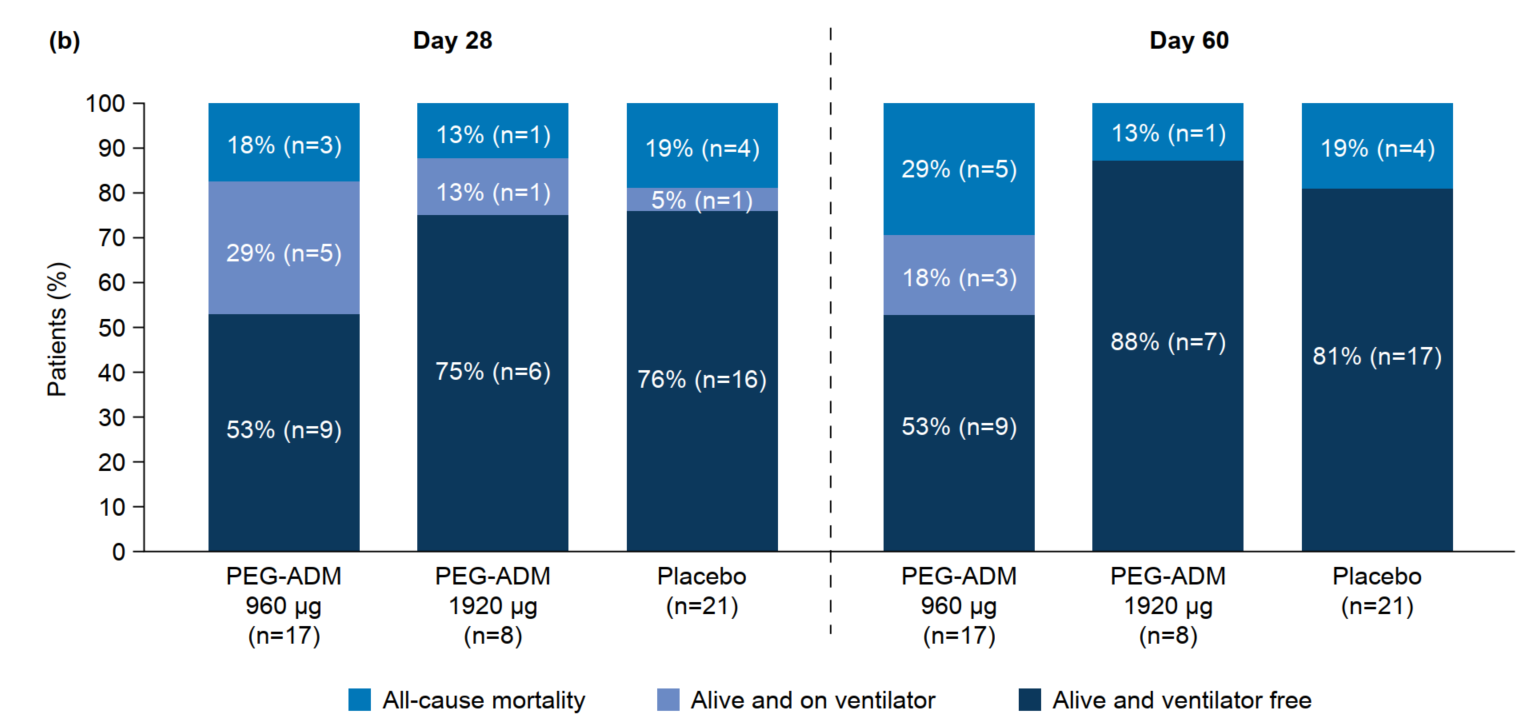


PEG, polyethylene glycol; PEG‑ADM, PEGylated adrenomedullin.

**Supplementary Table 1**. Full inclusion and exclusion criteria

| **Inclusion criteria** | **Exclusion** |
| --- | --- |
| - ≥18 years of age at the time of inclusion into study - Invasively mechanically ventilated ARDS patients (diagnosed according to Berlin definition of ARDS, including PEEP of ≥5 cm H2O, X-ray [or CT scan] indicative of ARDS: bilateral opacities not fully explained by cardiac failure, fluid overload, lobar/lung collapse, effusions or nodules). - Initial diagnosis of mild, moderate, or severe ARDS prior to study inclusion, with acute onset of ARDS within 1 week after suspected trigger factor of:   - Pneumonia   - Aspiration   - Sepsis   - Pancreatitis - Before randomisation, hypoxaemia with PaO_2_:FiO_2_ ≤300 mmHg continuously observed for a period of ≥4 hours (with values of ≥2 arterial blood gas analyses during that time, with the last value obtained timely [generally ≤3 hours] before randomisation), under ventilation with minimum PEEP ≥8 cm H_2_O (and considering the recommended ventilation strategy during this period) - Time from first meeting the last diagnostic ARDS criterion (Berlin criteria) to randomisation must be ≤48 hours - For Study Part A: ARDS patients for whom measurements of extra-vascular lung water are regarded as medically indicated by the treating physician, and these measurements are planned as part of their clinical care, from Study Day 1 up to Study Day 7 (if then still intubated) - Male and non-pregnant female ^a^ - Informed consent of capable patient or, in case of patient being incapable of giving informed consent, consent for study inclusion will be sought according to applicable laws and regulations | - Any value of a PaO_2_:FiO_2_ ratio >300 mmHg within a time interval of 4 hours before randomisation - Rescue therapy (e.g. inhalation of nitric oxide gas and/or inhalation of prostacyclin analogues, or ECMO/ECCO_2_R) already initiated at screening and/or Study Day 1 (prior to first dose of the study intervention)^b^ - Moribund participants not expected to survive 24 hours (clinical decision) - Expected duration of invasive mechanical ventilation less than 48 hours (clinical decision) - History of co-morbidities requiring long-term/home oxygen use (e.g. severe chronic obstructive pulmonary disease, pulmonary fibrosis) or non-invasive ventilation (except for sleep apnoea management), or making weaning per se improbable (e.g. ALS, muscular dystrophy) - Smoke inhalation injury, extensive burns, or trauma/head injury as concomitant condition - History of pneumectomy, lung lobectomy, or lung transplant - Diffuse alveolar haemorrhage from vasculitis - Current lung malignancy (including lung metastasis), or other malignancy requiring chemotherapy or radiation within the previous month - Chronic kidney disease with a history of renal replacement therapy (e.g. dialysis) - Chronic liver disease Child-Pugh Class C - Chronic heart failure (New York Heart Association class IV) - Known hypersensitivity to polyethylenglycol (Macrogol) - Diagnosis of COVID-19 pneumonia within 6 weeks prior to study inclusion ^c^ - Plan to participate or past participation (within 30 days prior to Study Day 1) in other interventional studies involving pharmacological interventions, or biological or cell therapy interventions ^d^ |

^a^ As the study population was critically ill, invasively mechanically ventilated and hence expected to be abstinent from sexual intercourse during the treatment period, the need for contraception advice during this period was recognised to be very unlikely. At the discretion of the investigator, advice to use adequate contraceptives was given to participants who regained consciousness and may have been sexually active before 30 days after end of treatment.

^b^ As there is no approved pharmacotherapy for ARDS, there is also no common rescue

medicine available. Any rescue procedures that were chosen at the discretion of the treating physician and according to published guidelines and the practice at the respective clinical site were recorded in the eCRF and were expected to include, for example, inhalation of nitric oxide gas, inhalation of prostacyclin analogues, airway pressure release ventilation, high frequency ventilation, or initiation of ECMO/ECCO_2_R. Use of systemic glucocorticoids has been previously considered as ARDS intervention [6], but doses >1 mg/kg methylprednisolone (or equivalent) were also regarded as a rescue therapy for the purpose of this study. If any other rescue procedures were initiated with the goal to improve the condition, investigators were required to document the respective rescue procedure in the eCRF.

^c^ History of SARS-CoV-2 infection (positive test based on nucleic acid amplification technology or positive antigen test) without COVID-19 pneumonia did not exclude patients.

^d^ Participation in other interventional studies that study modifications of supportive care regimens or similar objectives may be possible if the intervention in question was regarded not to endanger outcome assessments or supportive care recommendations described in the present study protocol. The investigator and sponsor were required to assess the implications of such studies; if no mutual agreement was reached, the patient was to be excluded.

ALS, amyotrophic lateral sclerosis; ARDS, acute respiratory distress syndrome; COVID-19, coronavirus disease 2019; CT, computed tomography; ECCO_2_R, extracorporeal CO_2_ removal; ECMO, extracorporeal membrane oxygenation; eCRF, electronic case report form; ECMO, extracorporeal membrane oxygenation; PEEP, positive end expiratory pressure.

**Supplementary Table 2**. Power calculations for Part A

|  | **Geometric mean ELVWi after treatment (mL/kg)** | | **Mean relative change in oxygenation index** | | **Change in mean npSOFA score after treatment** | | **Probability of achieving a ‘go’ decision** |
| --- | --- | --- | --- | --- | --- | --- | --- |
|  | **PEG-ADM** | **Placebo** | **PEG-ADM** | **Placebo** | **PEG-ADM** | **Placebo** |  |
| Scenario 1 | 7 | 12 | 40% | 14% | 0 | 0 | 98% |
| Scenario 2 | 12 | 12 | 14% | 14% | 0 | 0 | 16% |

EVLWi, extravascular lung water index; npSOFA, non-pulmonary Sequential Organ Failure Assessment; PEG, polyethylene glycol; PEG-ADM, PEGylated adrenomedullin.

**Supplementary Table 3**. Ventilator-free days and length of hospital and intensive care stay to Day 28

| **Mean (SD) days** | **PEG-ADM 960 μg**  **(n=29)** | **PEG-ADM 1920 μg**  **(n=30)** | **Placebo**  **(n=29)** |
| --- | --- | --- | --- |
| Ventilator-free days | 9.2 (10.4)^a^ | 10.7 (9.4)^b^ | 9.8 (8.1) |
| Length of hospital stay | 21.6 (6.9) | 21.5 (8.0) | 21.9 (7.9) |
| Length of intensive care stay | 18.0 (8.1) | 16.0 (7.5) | 17.1 (8.1) |

^a^p=0.394 vs. placebo; ^b^p=0.343 vs placebo.

PEG, polyethylene glycol; PEG-ADM, PEGylated adrenomedullin; SD, standard deviation.

**Supplementary Table 4**. Fluid management

| **CVP^a^, mmHg** | **PAOP^a^, mmHg** | **Mean artery pressure ≥60 mmHg and off vasopressors for ≥12 hours** | |
| --- | --- | --- | --- |
|  |  | **Average urine output <0.5 mL/kg/hour** | **Average urine output ≥0.5 mL/kg/hour** |
| >8 | >12 | Furosemide^b^ (reassess in 1 hour) | Furosemide^b^ (reassess in 4 hours |
| 4–8 | 8–12 | Give fluid bolus as quickly as possible  (reassess in 1 hour) |  |
| <4 | <8 |  | No intervention  (reassess in 4 hours) |

^a^If available.

^b^Recommended furosemide dosing: start with 20 mg bolus or 3 mg/hour infusion, or last known effective dose. Double each subsequent dose until goal achieved (oliguria reversal or intravascular pressure target) or maximum infusion rate of 24 mg/hour or 160 mg bolus is reached. Do not exceed 620 mg/day. If participant has heart failure, treatment with dobutamine might also be considered.

CVP, central venous pressure; PAOP, pulmonary artery occlusion pressure.

**Supplementary Table 5**. Subgroup analysis due to severity of ARDS


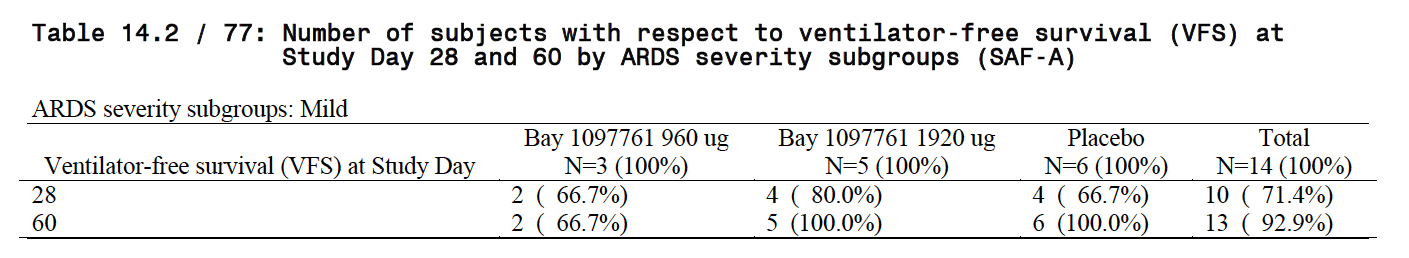


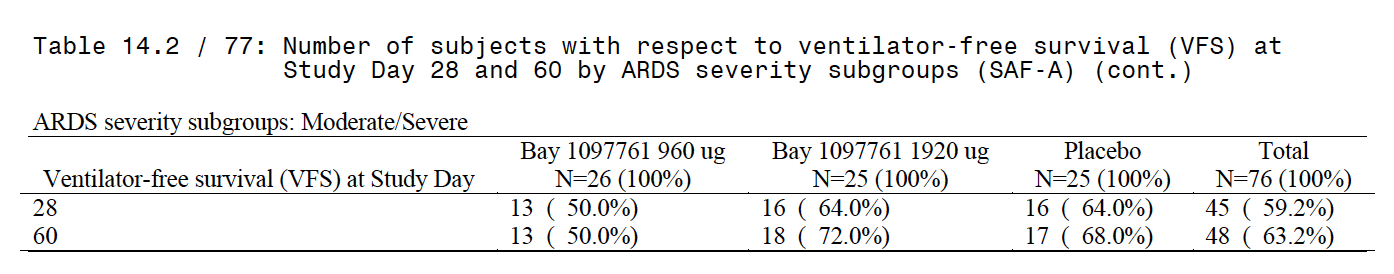


**Supplementary Table 6**: Oxygenation Index (day 1-28)


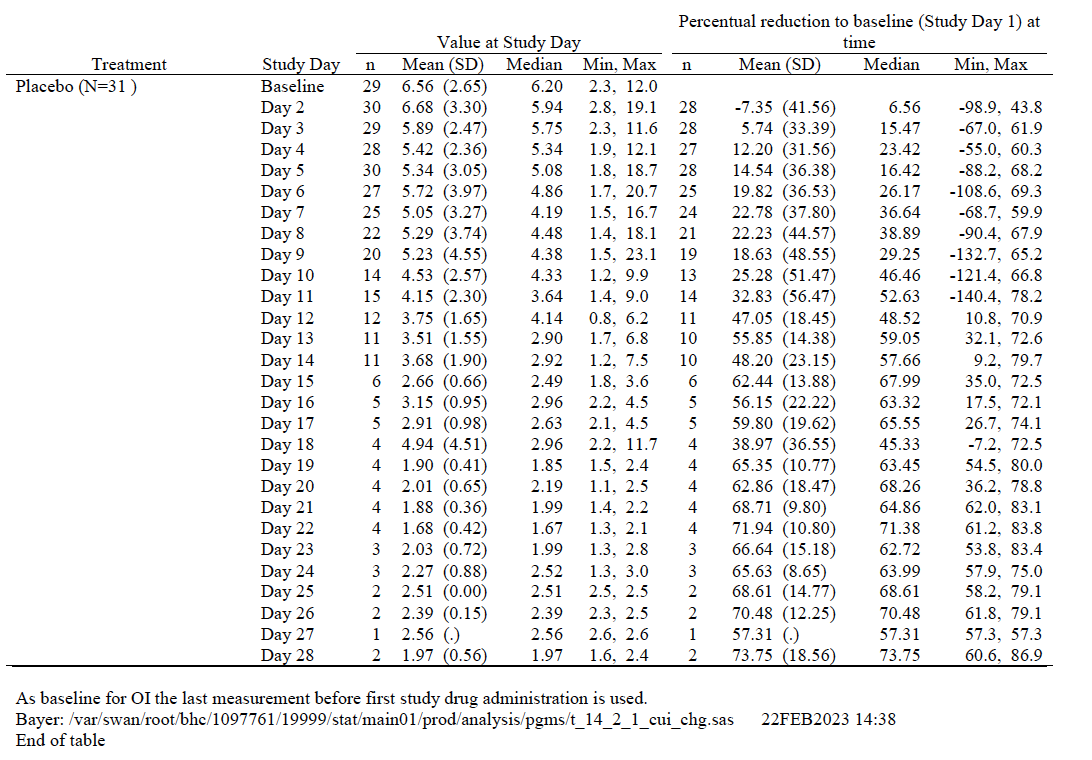


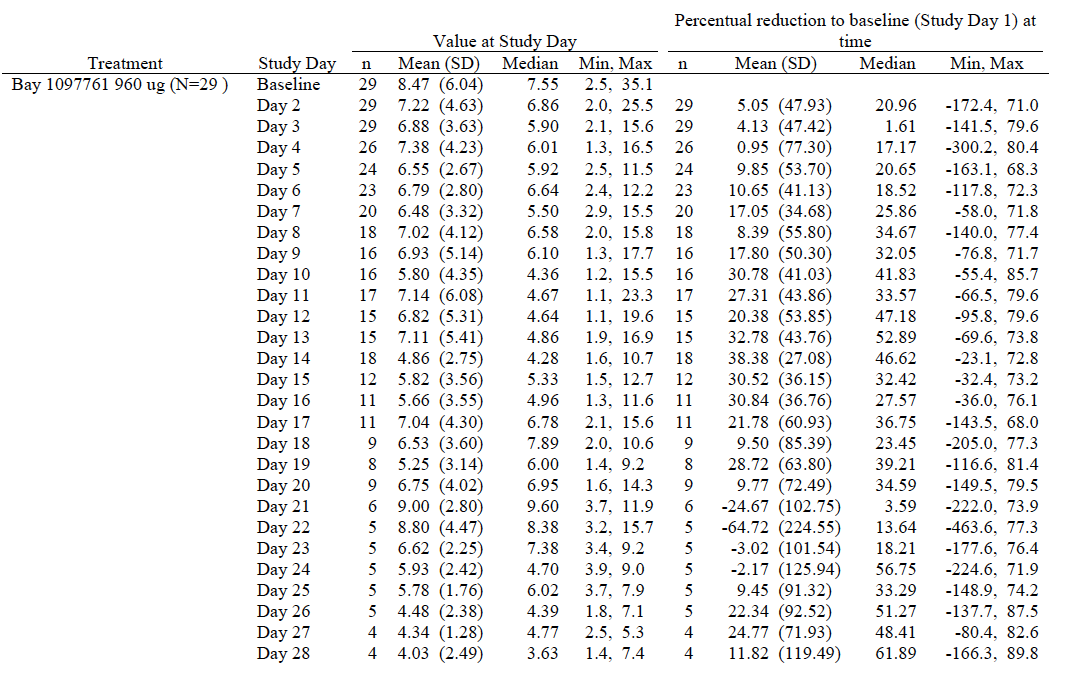


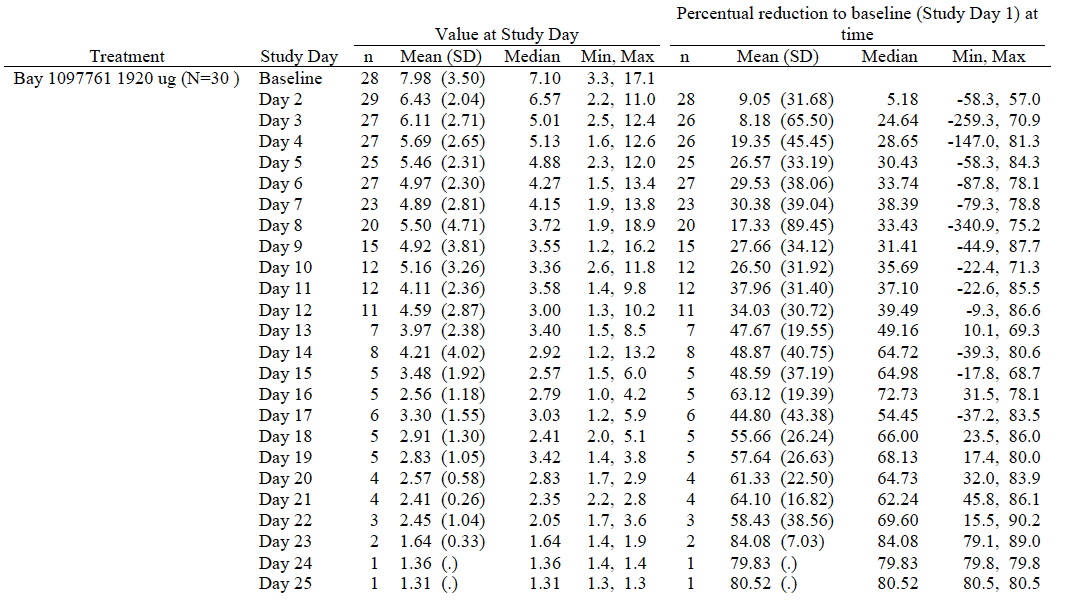


**Supplementary Table 7**: HCO3- from day 1 to 28


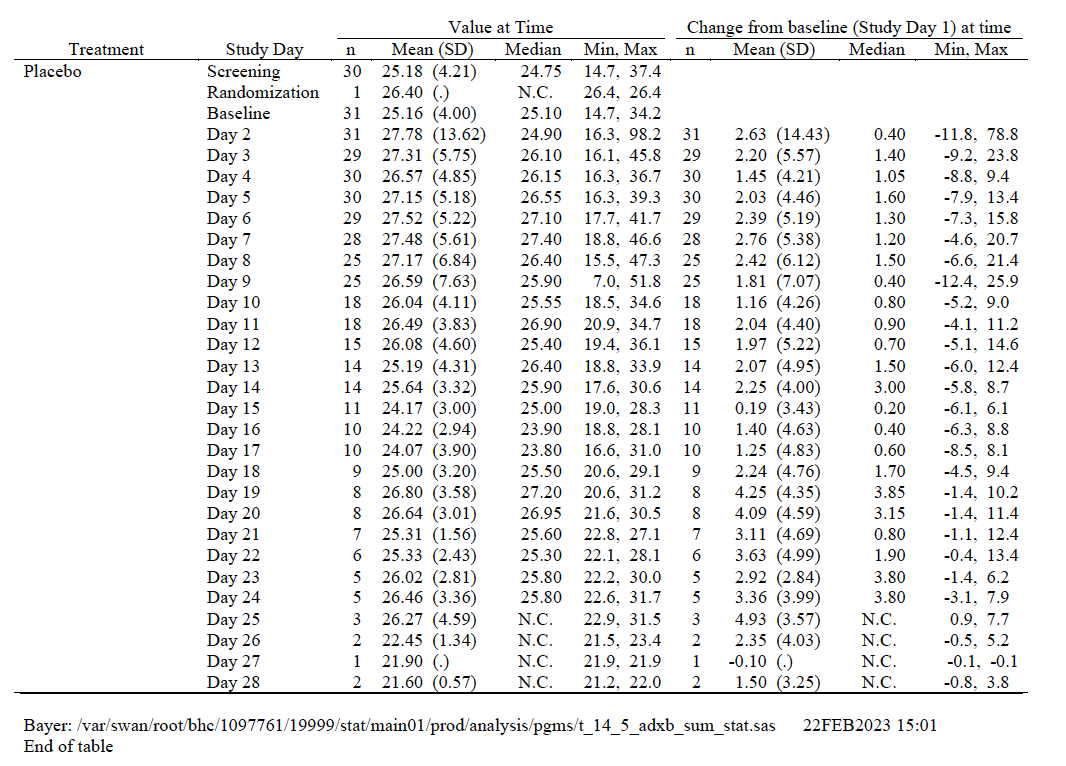


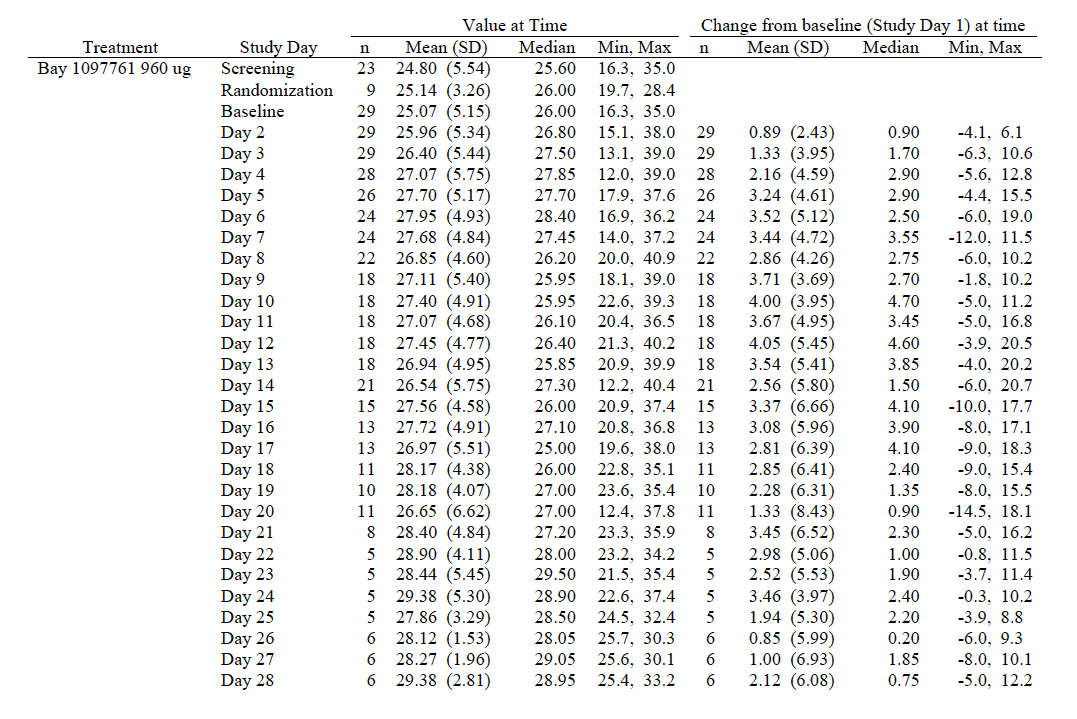


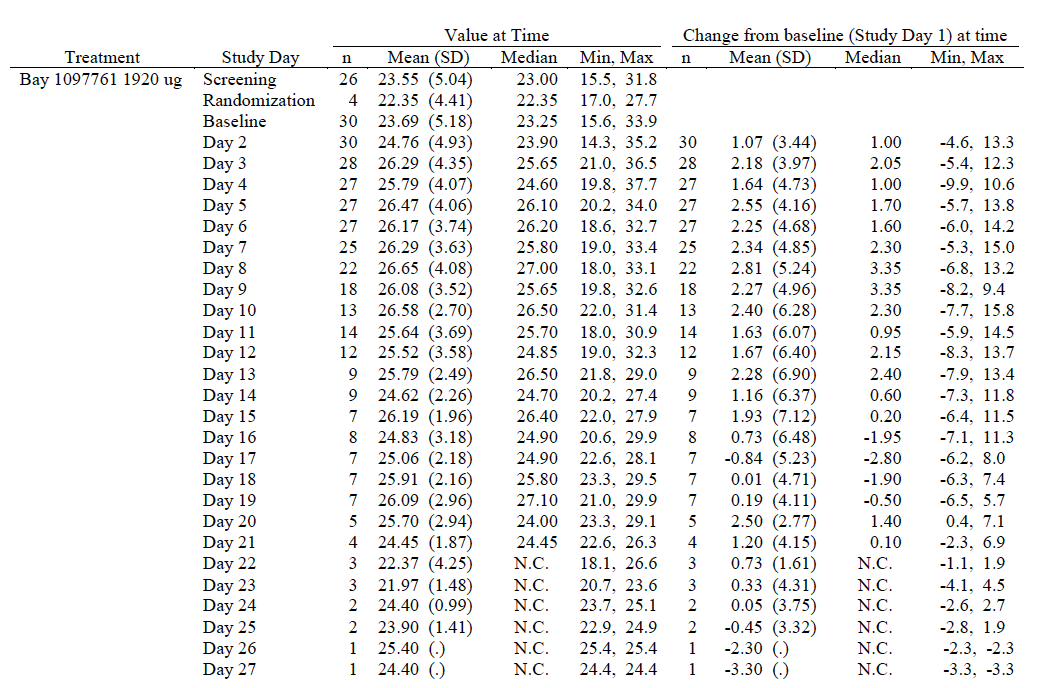


### Supplemental references

1. Huang DT, Angus DC, Moss M, et al. Design and rationale of the reevaluation of systemic early neuromuscular blockade trial for acute respiratory distress syndrome. Ann Am Thorac Soc 2017;14(1):124–33. doi:10.1513/AnnalsATS.201608-629OT
2. Acute Respiratory Distress Syndrome Network, Brower RG, Matthay MA, et al. Ventilation with lower tidal volumes as compared with traditional tidal volumes for acute lung injury and the acute respiratory distress syndrome. N Engl J Med 2000;342(18):1301–8. doi:10.1056/NEJM200005043421801
3. Linares-Perdomo O, East TD, Brower R, Morris AH. Standardizing predicted body weight equations for mechanical ventilation tidal volume settings. Chest 2015;148(1):73–8. doi:10.1378/chest.14-2843
4. Grissom CK, Hirshberg EL, Dickerson JB, et al. Fluid management with a simplified conservative protocol for the acute respiratory distress syndrome*. Crit Care Med 2015;43(2):288–95. doi:10.1097/CCM.0000000000000715
5. Famous KR, Delucchi K, Ware LB, et al. Acute respiratory distress syndrome subphenotypes respond differently to randomized fluid management strategy [published correction appears in Am J Respir Crit Care Med 2018;198(12):1590] [published correction appears in Am J Respir Crit Care Med 2019;200(5):649]. Am J Respir Crit Care Med 2017;195(3):331–8. doi:10.1164/rccm.201603-0645OC
6. Annane D, Pastores SM, Rochwerg B, et al. Guidelines for the diagnosis and management of critical illness-related corticosteroid insufficiency (CIRCI) in critically ill patients (Part I): Society of Critical Care Medicine (SCCM) and European Society of Intensive Care Medicine (ESICM) 2017. Crit Care Med 2017;45(12):2078–88. doi:10.1097/CCM.0000000000002737
